# Mitotic chromosomes

**DOI:** 10.1016/j.semcdb.2021.03.014

**Published:** 2021-09

**Authors:** James R. Paulson, Damien F. Hudson, Fernanda Cisneros-Soberanis, William C. Earnshaw

**Affiliations:** a Department of Chemistry, University of Wisconsin Oshkosh, 800 Algoma Boulevard, Oshkosh, WI 54901, USA; b Murdoch Children's Research Institute, Royal Children's Hospital, Parkville, VIC 3052, Australia; c Wellcome Trust Centre for Cell Biology, ICB, University of Edinburgh, Michael Swann Building, King's Buildings, Max Born Crescent, Edinburgh EH9 3BF, Scotland, UK

**Keywords:** Chromosome, Scaffold, Condensin, Cohesin, Topoisomerase IIα, KIF4

## Abstract

Our understanding of the structure and function of mitotic chromosomes has come a long way since these iconic objects were first recognized more than 140 years ago, though many details remain to be elucidated. In this chapter, we start with the early history of chromosome studies and then describe the path that led to our current understanding of the formation and structure of mitotic chromosomes. We also discuss some of the remaining questions. It is now well established that each mitotic chromatid consists of a central organizing region containing a so-called “chromosome scaffold” from which loops of DNA project radially. Only a few key non-histone proteins and protein complexes are required to form the chromosome: topoisomerase IIα, cohesin, condensin I and condensin II, and the chromokinesin KIF4A. These proteins are concentrated along the axis of the chromatid. Condensins I and II are primarily responsible for shaping the chromosome and the scaffold, and they produce the loops of DNA by an ATP-dependent process known as loop extrusion. Modelling of Hi-C data suggests that condensin II adopts a spiral staircase arrangement with an extruded loop extending out from each step in a roughly helical pattern. Condensin I then forms loops nested within these larger condensin II loops, thereby giving rise to the final compaction of the mitotic chromosome in a process that requires Topo IIα.

## 1 Mitotic chromosomes and the chromosome structure problem

The discovery of eukaryotic chromosomes arose from studies of cell division in the second half of the 19th century and was made possible by the development of improved compound microscopes by Zeiss in Jena [1]. Edouard Balbiani clearly observed chromosomes and described and illustrated metaphase plates in 1861, though he misinterpreted what they were [2], [3], [4]. Edouard van Beneden saw mitotic chromosomes and called them “bâtonnets” (little rods) when he described cell division in 1875. German scientists also referred to them as little rods (“Stäbchen”) or as “Fäden” (threads) [5].

**Fig. 1:**
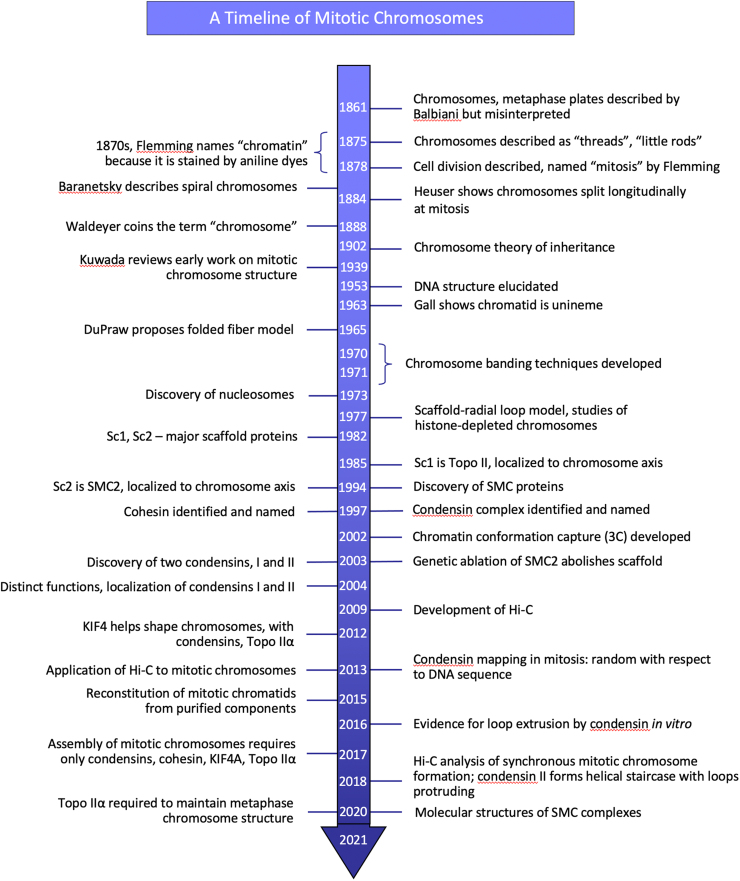
Selective timeline of key events in mitotic chromosome studies. For simplicity, references are omitted from the figure, but these are given in the relevant sections of the text.

Walther Flemming’s very careful observations of cell division in the 1870s showed that nuclei do not divide by simple fission, as had been widely believed, but instead undergo a transformation in which the “threads” appear. He therefore named the process *mitosis*, from the Greek *μίτος* (*mitos*, “warp thread”) and described its basic features much as we know them today [5], [6], although he missed the fact that the chromosomes split in half longitudinally, with one sister chromatid going to each spindle pole. This was corrected a few years later by Emil Heuser [7]. Flemming also adapted the use of dyes from bacteriology, and this led him to call the threads and nuclear material “chromatin” because of their ability to take up stain. Based on this, Waldeyer later named the threads “chromosomes” [8]. The importance of chromosomes became clear when Sutton [9] observed that their behavior in mitosis and meiosis corresponds to the Mendelian behavior of genes. Together, these observations led to the chromosome theory of inheritance. A timeline of key landmarks leading to our current understanding of mitotic chromosome structure is presented in Fig. 1.

We now know that the hereditary material is DNA and that a mitotic chromosome consists of DNA complexed with proteins. Each chromatid in a mitotic chromosome is unineme; that is, it contains a single very long DNA double helix that wends its way from one end (telomere) of the chromatid to the other. This was first established by Joe Gall’s studies on fragmentation of lampbrush chromosomes [10] and other observations (reviewed by [11]) and has since been thoroughly confirmed by genome sequencing and physical mapping of chromosomes. These chromosomal DNA molecules are highly compacted in length. For example, the largest human mitotic chromosome is about 8–10 µm long but contains a DNA molecule 8.5 cm long (2.5 × 10^8^ base pairs), indicating a 10^4^-fold lengthwise compaction [12]. During interphase, the same chromosome must also be compacted about 8500-fold to fit into a typical cell nucleus (about 10 µm in diameter), so the difference in chromatin compaction between mitotic and interphase chromosomes is actually relatively subtle. Chromatin is typically estimated to be 2–3-fold more compact in mitosis [13], [14], [15], but that may be an overestimate. A recent electron microscopy tomographic analysis finds an average chromatin concentration of 30 ± 10% (i.e., the percentage of the volume occupied by chromatin) in interphase nuclei compared with 42 ± 2.5% in mitotic chromosomes [16].

Compaction of the chromosomal DNA in both interphase and mitosis is explained in part by formation of the chromatin fiber, in which the DNA is wound around core histones to form a string of disk-shaped nucleosomes [17]. Under appropriate salt conditions in vitro, this “string of beads” is compacted further by linker histones into a fiber about 30 nm in diameter [18]. For a long time, it was thought that irregular 30 nm fibers also constitute the bulk of chromatin in vivo, both in interphase and mitosis. Evidence for this came from electron microscopy (e.g., [19], [20], [21]) as well as low-angle x-ray diffraction (e.g., [22], [23]). However, a general consensus is now emerging that canonical 30 nm fibers may be rare, except in some specialized cells such as avian erythrocytes [16], [24], [25]. High resolution tomographic imaging of chromosomal DNA in interphase nuclei instead reveals a disordered granular primary chain 5–24 nm in diameter [16], with higher-order structures resembling clusters or “clutches” of nucleosomes [26], [27], often referred to as chromatin condensates [28], [29]. Chromatin density is lowest in the nuclear interior and highest in the heterochromatin near the nuclear lamina. The chromatin chain appears to be more flexible in mitotic chromosomes, and this allows nucleosomes to pack more closely together, but still with no regular organization of nucleosomal fibers [16]. Regular fibers greater than 100 nm in diameter are rare [30], although chromatin domains approximately 200 nm diameter, described in earlier conventional electron microscopy studies [31], [32], [33], [34], have been observed in interphase and mitotic chromosomes [35], [36]. Others have referred to this concentrated phase of nucleosomes in mitotic chromosomes as a “polymer melt” [37]. Importantly, although chromatin can undergo liquid-liquid phase separation (LLPS - [38]) under certain conditions [28], careful studies suggest that the chromatin behaves as a solid that may organize liquid-like domains of associated proteins and RNAs around itself [29].

As mentioned above, the difference in chromatin compaction between interphase and mitosis is subtle, as are the differences between interphase and mitotic chromatin fibers. By contrast, the morphological changes between interphase and mitotic chromosomes are dramatic indeed! How do these striking changes come about?

In this chapter, we review some of the progress that has been made in understanding the structure of mitotic chromosomes since they were first observed more than 140 years ago. The chromatid has been variously described as a linear array of bead-like granules (chromomeres) [39], [40], a rod-like “unit fiber” [41], a coiled filament (chromonema) [42], [43], a brush-like aggregate of loops [21], [44], or a mass of disordered chromatin (polymer melt) [19], [37], [45], and paradoxically all these models have had support from microscopy. But as our understanding has advanced, the paradox has begun to be resolved. The appearance of chromosomes in the microscope presumably reflects their intrinsic structure, but evidently can vary because they are elastic [46], [47], [48] and structurally “metastable” (i.e., can undergo rapid structural transformations in response to their environment or as a result of preparation techniques). One common example, not yet fully understood, is the transition from paired rod-like sister chromatids to paired helical spirals [43], [49]. Whether this metastable property of mitotic chromosomes reflects a “stressed substructure” [50], [51], [52], [53] remains to be determined. For selected reviews on mitotic chromosome structure, see [11], [12], [54], [55], [56], [57], [58], [59], [60], [61], [62], [63], [64], [65], [66], [67], [68].

A recent study using correlative light and serial block face scanning electron microscopy coupled with three-dimensional reconstruction of whole mitotic cells yielded the surprising result that up to 40% of the volume of mitotic chromosomes is not chromatin. Instead, it consists of a layer of proteins and RNAs, organized by the protein Ki-67, that coats the surface of chromosomes from prometaphase through anaphase [69], [70]. This mitotic chromosome periphery compartment (MCPC) is largely made up of highly abundant components derived from nucleoli, including assembly intermediates of large and small ribosomal subunits (for reviews see [66], [71]). The role, if any, of Ki-67 in shaping the mitotic chromosomes appears to be relatively subtle and may be non-essential [72], [73], [74].

## 2 Early models: chromomeres and the coiled chromonema

Early attempts to explain the structure of mitotic chromosomes gave rise to two main types of models. In the first, the mitotic chromatid was seen as a string (spireme) of chromomeres (bead-like granules) [40]; in the second, it was thought to consist of a helix or spiral [43]. It should be noted that these ideas were based solely on light microscopy, and both originated before anything was known about the composition of chromosomes or the nature of DNA.

Early researchers described the denser regions seen along the length of mitotic or meiotic chromosomes as chromomeres by analogy with the dark bands seen in giant polytene chromosomes [39], [40]. However, chromomeres were only vaguely defined, they were generally seen only in prophase chromosomes (not prometaphase or metaphase), and their structural significance, if any, was never clear. The term “chromomere” is still used, but only rarely in relation to mitotic chromosomes (e.g., [75]).

Observations of helical chromosomes, first by Baranetzky [76] and subsequently by many other authors, led to the notion of a “coiled chromonema” (i.e., coiled thread or fiber) or spireme as the basic structure of the chromatid [40], [42], [77], [78]. A comprehensive review of mitotic chromosome structure published in Japan in 1939 by Kuwada [43] focused on this idea and by the 1950s this was the dominant model. Obvious spirals and even smaller coils within macro-spirals are sometimes seen by light microscopy, particularly in plants with large chromosomes such as *Tradescantia* and *Lilium*, e.g., [43], [79], [80], [81], [82], [83], but they have also been seen (rarely) in mammalian cells [49], [84], [85]. Indeed, once the structure of DNA was known, helical models of chromosomes became more plausible. It made sense that the DNA double helix might twist into a supercoil which in turn could super-twist into shorter, fatter solenoidal helices as mitotic chromosomes formed [31], [32], [34]. On the other hand, in the absence of knowledge of topoisomerases, these models required elaborate mechanisms to permit topological disentanglement and segregation of sister chromatids at anaphase (see for example [43]).

At one point it was proposed that each chromatid consists of a coiled “unit fiber” about 0.4 µm in diameter [41]. However, subsequent work indicated that the extraordinarily dense unit fiber is an artefact of shearing during the preparation of chromosomes [86], [87], [88]. In fact, each pair of sister chromatids gives rise to a single unit fiber [87]. How paired sister chromatids can undergo such a drastic reorganization remains a mystery but is one of the observations suggesting that mitotic chromosome structure is somehow metastable. Possibly, the chromosome as a whole becomes more susceptible to shear when connections within the chromosome scaffold (see below) are weakened.

As discussed below, it is now clear that each chromatid of a mitotic chromosome does not simply consist of a hierarchical series of coils. Instead, and despite some recent claims to the contrary based on very detailed analysis by light microscopy [52], modelling of Hi-C data from vertebrate chromosomes undergoing synchronous mitotic entry clearly argues that each sister chromatid is organized around a helical array of condensin II, from which loops project radially, as though from the steps of a spiral staircase [89]. Those and previous Hi-C experiments [90] appear to confirm and extend the radial loop model proposed by Laemmli and co-workers (see below), who had observed that in some histone H1-depleted and decondensed chromosomes the chromosome scaffold component topoisomerase IIα follows a helical path, as seen by immunofluorescence staining [91].

## 3 The folded fiber model of chromosome structure

The folded fiber model of Ernest DuPraw [19], [45], [92] was the first model of mitotic chromosome organization that did not include either chromomeres or hierarchical spirals, and it was also the first model based on electron microscopy rather than just light microscopy. “Whole mount” mitotic chromosomes were spread on a liquid surface, picked up on grids and critical point dried. These chromosomes retained their normal overall shape, and the chromatids appeared bushy with segments of chromatin fibers protruding at the surface. Interestingly, DuPraw occasionally referred to the folded fiber as “looping out” at telomeres and compared this to the “spinning out” of DNA in the loops of diplotene lampbrush chromosomes and the “puffs” of polytene chromosomes. However, he never suggested loops as a general feature of the folded fiber. In a few cases, DuPraw interpreted his micrographs as showing coiled chromatids (e.g., [92]), but these were seen only rarely and coiling was not an integral aspect of his folded fiber model.

DuPraw proposed that the fibers were folded randomly and often longitudinally in chromatids, but these ideas were confuted by the development of chromosome banding techniques, (e.g., [93], [94], [95], [96], [97], [98], [99]). The bands consistently traverse a chromatid from side to side, suggesting that the fiber mainly lies perpendicular to the chromosome axis rather than running parallel to it [98]. The explanation for these banding patterns remains somewhat mysterious, though the most likely explanation is that they reflect large scale trends in the G:C and A:T content of the DNA (e.g., the “isochores” of Bernardi [100], [101], [102]).

The advent of chromosome banding techniques also showed that mitotic chromosome structure, at least at the scale of banding patterns, is quite reproducible. Comparing karyotypes of closely related species by banding (e.g., humans and other primates; [103]) or chromosome painting [104], shows that mitotic chromosome structure is largely preserved over millions of cell generations. It is hard to imagine how random folding, as postulated in DuPraw’s model, could produce such consistency of structure.

## 4 Non-histone proteins and the scaffold-radial loop model

DuPraw’s electron micrographs and his folded fiber model represented an important advance, but like the chromomere and coiled chromonema models, his model did not envision any structural elements in chromosomes other than DNA and histones. Yet it was never clear how histones alone would be able to maintain stable chromosome morphology over millions of cell divisions.

A major breakthrough was made when Laemmli and co-workers removed bulk histones from isolated chromosomes and studied the structure and composition of what remained. Treatment of isolated prometaphase chromosomes with high salt (2 M NaCl), or with polyanions in the presence of low salt, solubilized the histones and produced residual DNA-containing structures sedimenting at 4000–7000 S and essentially devoid of histones. These structures were disrupted by treatment with urea, SDS, or chymotrypsin, but not by RNase or high salt, suggesting that they were held together by non-histone proteins [105].

**Fig. 2:**
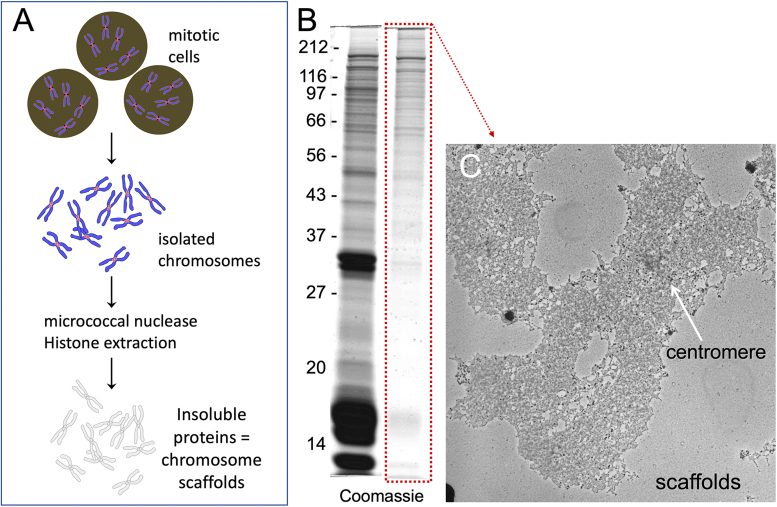
Isolation and characterization of the mitotic chromosome scaffold fraction. (A) General flow-chart for the isolation of mitotic chromosome scaffolds. (B) SDS polyacrylamide gel of mitotic chromosomes (lane 1) and the scaffold fraction isolated by treatment with nucleases and 2 M NaCl (lane 2) from chicken MSB-1 cells [127]. (C) Human (HeLa) mitotic chromosome scaffold isolated at low ionic strength with dextran sulphate/heparin and centrifuged onto an electron microscope grid [87].

Electron microscopy of surface-spread histone-depleted chromosomes showed a central structure with the general shape of a metaphase chromosome, from which DNA extended as loops [44]. This central structure was termed the chromosome “scaffold”. The scaffold evidently involved non-histone proteins since it could be visualized by silver staining in intact chromosomes or in chromosome spreads after DNase and high salt treatment but was destroyed by treatment of those spreads with urea or a mixture of proteases [106]. A scaffold fraction rich in non-histone proteins and with almost all DNA removed could be prepared from isolated mitotic chromosomes by digesting the DNA first and then depleting the chromosomes of histones [107]. A description of the procedure and some characterization of the scaffold fraction is presented in Fig. 2.

**Fig. 3:**
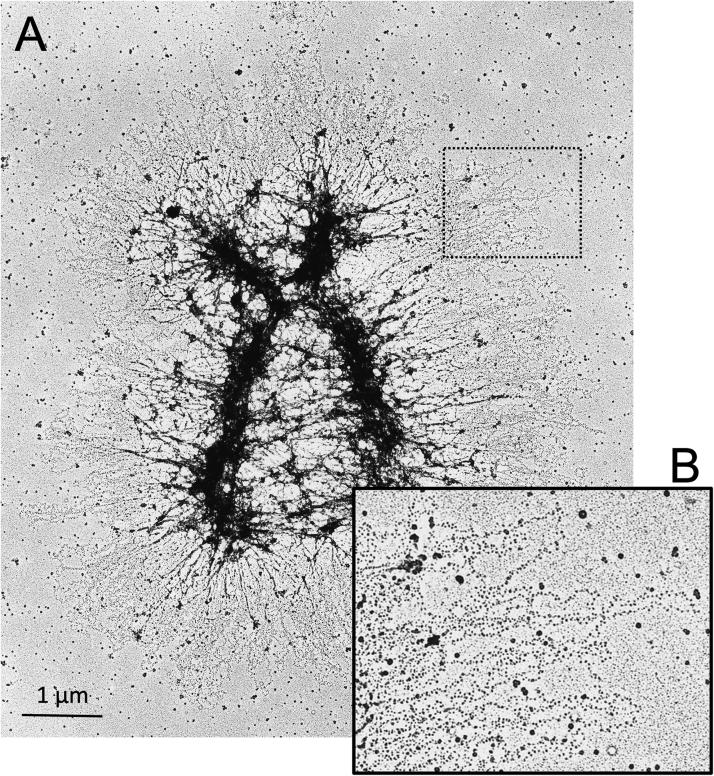
Human mitotic chromosome showing radial loops with nucleosomes. (A) Chromosomes were isolated from HeLa cells, expanded at low ionic strength in the absence of divalent cations using TEEN buffer, centrifuged onto an electron microscope grid and then treated with aqueous uranyl acetate (which caused the chromatin not adherent to the carbon film to collapse back onto the chromatid axes) [87]. (B) Enlarged view of the boxed area. Radial loops with abundant nucleosomes are clearly seen.

These results led to the Scaffold-Radial Loop Model of mitotic chromosome structure [44], [108], which proposed that a scaffold of non-histone proteins is responsible for the basic shape of mitotic chromosomes and organizes the DNA into loops along the length of each chromatid. In thin-sections of isolated chromosomes, Marsden and Laemmli observed a radial distribution of chromatin loops projecting outward from the central axis of each chromatid [21], and Adolph obtained similar results using thin-sections of permeabilized cells [109], [110]. Marsden and Laemmli estimated the length of these loops, and assuming the existence of a 30 nm chromatin fiber in the isolated chromosomes, their results indicated a loop size of around 80 kb [21]. This agrees remarkably well with DNA loop lengths measured by Paulson and Laemmli [44], measurements of HeLa cell nucleoids [111] and recent estimates based on Hi-C ([89] see Section 9). The presence of loops and a central organizing structure was subsequently confirmed in meiotic prophase chromosomes prepared by the Miller spreading technique [112], [113], [114] and by swelling and shrinking mitotic chromosomes that had been centrifuged onto electron microscope grids (Fig. 3) [87]. The latter studies also estimated a DNA loop size of around 80 kb, again assuming that the chromatin formed a 30 nm fiber in vitro.

The idea of loops emanating from a central axis was consistent with historic evidence for a loop organization of meiotic diplotene “lampbrush” chromosomes (e.g., [115], [116], [117], [118], [119]). Much later, loops were also resolved in transcriptionally active puffs of *Chironomus* polytene chromosomes [120]. To put these developments in context, several years before the discovery of the mitotic chromosome scaffold, Don Coffey and co-workers had proposed that nuclei in interphase cells were organized by a network of non-histone proteins that they termed the “nuclear matrix” [121], [122]. Despite years of sometimes bitter controversy (see below), we now know that non-histone proteins have major roles in organizing both the interphase genome and mitotic chromosomes (though they are not necessarily the same non-histone proteins in both cases).

## 5 The chromosome scaffold controversy

For many years, the existence of a chromosome scaffold was controversial. The dominant argument against it was that no one had observed a scaffold in “native” mitotic chromosomes (or a matrix in nuclei) by electron microscopy. It was argued that the scaffold/matrix was an artefact reflecting the precipitation of non-histone proteins under non-physiological salt conditions when the DNA was removed (see e.g. [123]). The use of the term “scaffold” may have been unfortunate, as many people interpreted the term in the sense of scaffolds used in building construction – i.e., as describing a solid rod-like structure upon which the chromosome was assembled [124]. Such robust rod-like structures had never been observed in electron micrographs. However, the original papers on histone-depleted chromosomes [44], [105], [107]) never claimed such a structure for the scaffold, instead describing it as “fibrous” and “a non-histone protein network”.

Over the years evidence has accumulated that the chromosome scaffold consists of specific proteins distributed axially, even in unextracted chromosomes. The precise organization of this scaffold remains a question of study, but evidence suggests that it consists of a network of connections [88] or a series of “spot-welds” [124], [125] that anchor (and likely create) the chromosome loops, rather than a rigid rod-like structure.

**Fig. 4:**
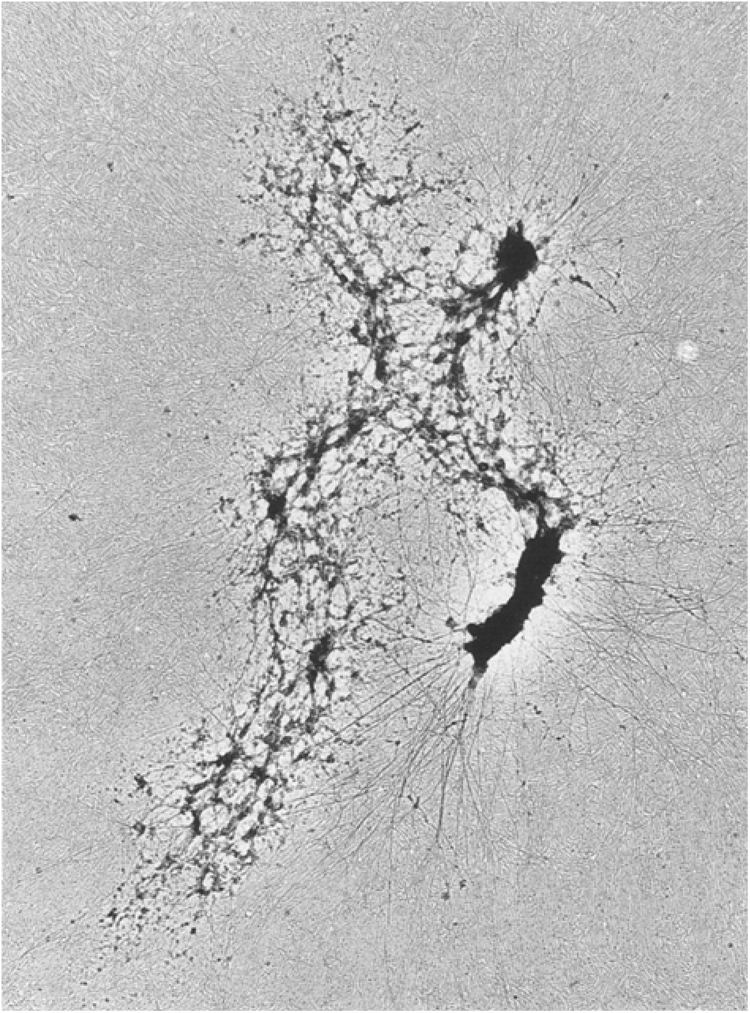
Scaffold region of a histone-depleted human mitotic chromosome. Chromosomes isolated from HeLa cells were depleted of histones by treatment with 2 M NaCl and prepared by Kleinschmidt spreading [460]. Note the open, network-like structure of the scaffold in the chromatid on the left. The dense “core” in the chromatid on the right and the “straps” emanating from it [126] are most likely due to collapse during dehydration and staining of DNA that was not completely adsorbed to the cytochrome c monolayer.

Among the electron micrographs of histone-depleted chromosomes published by Paulson [88], many show the scaffold as an open meshwork. However, some histone-depleted chromosomes were observed in which parts of the scaffold had an open network structure while other parts of the same scaffolds looked like solid cores. The latter showed features such as “straps” [126], indicating that the DNA had not completely adhered to the cytochrome c monolayer during spreading and had subsequently aggregated during the dehydration and staining steps. An example is shown in Fig. 4. It was suggested, therefore, that the open network-like appearance was more indicative of the underlying structure of the scaffold, and that the solid “chromosome cores” were produced artefactually [88].

(From [88] with permission.).

Subsequent immunoelectron microscopy of mitotic chromosomes that had been centrifuged onto electron microscope grids revealed that topoisomerase II was distributed throughout the central (axial) region of the chromatids as a series of dispersed spot-like foci [125]. The same study provided compelling evidence, based on a novel approach involving antibody crosslinking within isolated mitotic chromosomes, that topoisomerase II was concentrated at the base of the chromosomal loops with little or none stably associated with the radial loop DNA [125].

**Fig. 5:**
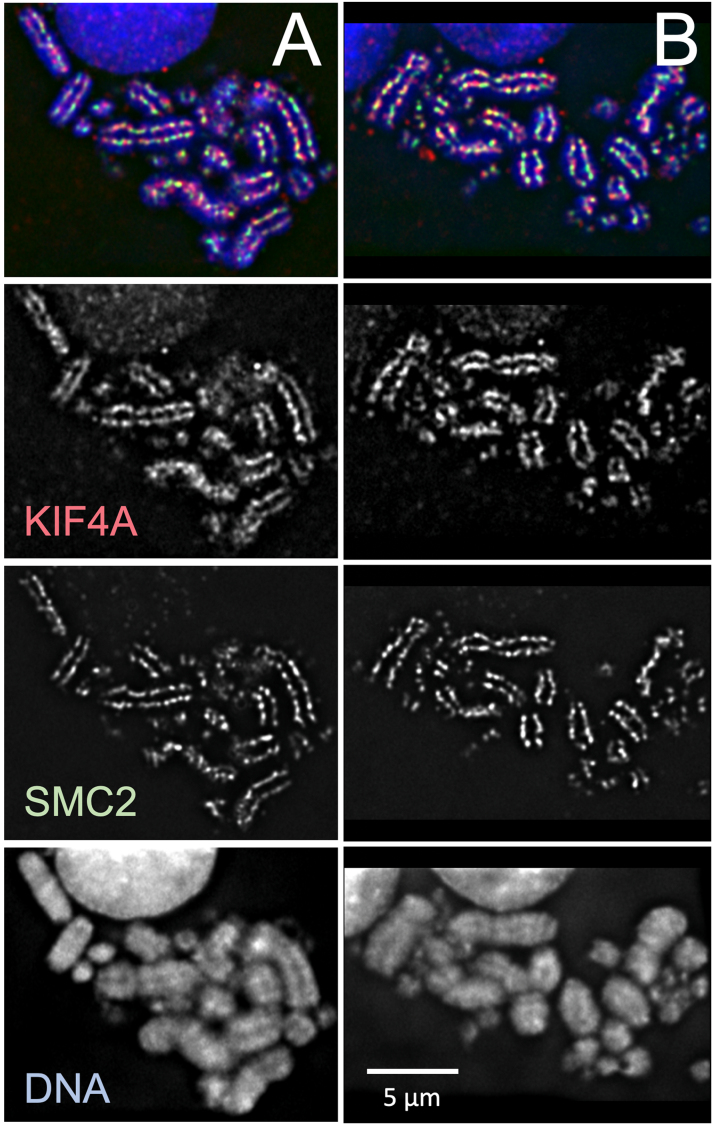
Localization of condensin and KIF4A to the chromatid axis in mitotic cells. (A,B) Two mitotic DT40 cells with conditional SMC2 knockout (SMC2^OFF^) stained for endogenous KIF4A (red) and expressing SMC2-TrAP (green - stained with anti-SBP antibody). Both proteins are localized to the axis of sister chromatids (DNA - blue). Unpublished images provided by Kumiko Samejima from [133].
• the fact that chromosomes can be swollen and then returned to their original size and morphology by manipulating the ionic strength [87], [162], suggesting that their structural integrity must involve a network of crossties [163];
• the fact that the reversibility of this swelling and therefore the structure of the network is dependent on condensin [132];
• the observation that genetic ablation of the condensin complex completely abolished the chromosome scaffold when isolated mitotic chromosomes were digested with nuclease and extracted with 2 M NaCl [132]. It is highly unlikely that removal of a single protein would cause all other chromosome-associated non-histone proteins to change their precipitation behavior.
• And finally, it has been shown that DNA can form chromosome-like structures by association with condensin complexes in a fractionated *Xenopus* egg extract system even without significant amounts of histones present [164].

Any concerns that the entire scaffold might be an artefact of specimen preparation were finally laid to rest by a series of further advances:

• the discovery of specific protein components of the scaffold, such as topoisomerase IIα [127], [128], SMC proteins [129] and the condensin complex [130], [131];
• the demonstration that these proteins are required for assembly of mitotic chromosomes [130], [131], [132], [133], [134], [135]. For reviews see [48], [55], [56], [57], [59], [60], [65], [66], [68], [136], [137], [138], [139], [140], [141], [142], [143], [144], [145], [146], [147], [148], [149], [150], [151], [152], [153], [154], [155], [156], [157], [158], [159], [160], [161];
• the demonstration that these proteins have an axial distribution even in unextracted chromosomes ([125], [128], [129], [130], and subsequently, too many references to list) (see e.g., Fig. 5);

Thus, after 40 years, it is no longer controversial to refer to a “chromosome scaffold” compartment running along the axes of sister chromatids.

**Fig. 6:**
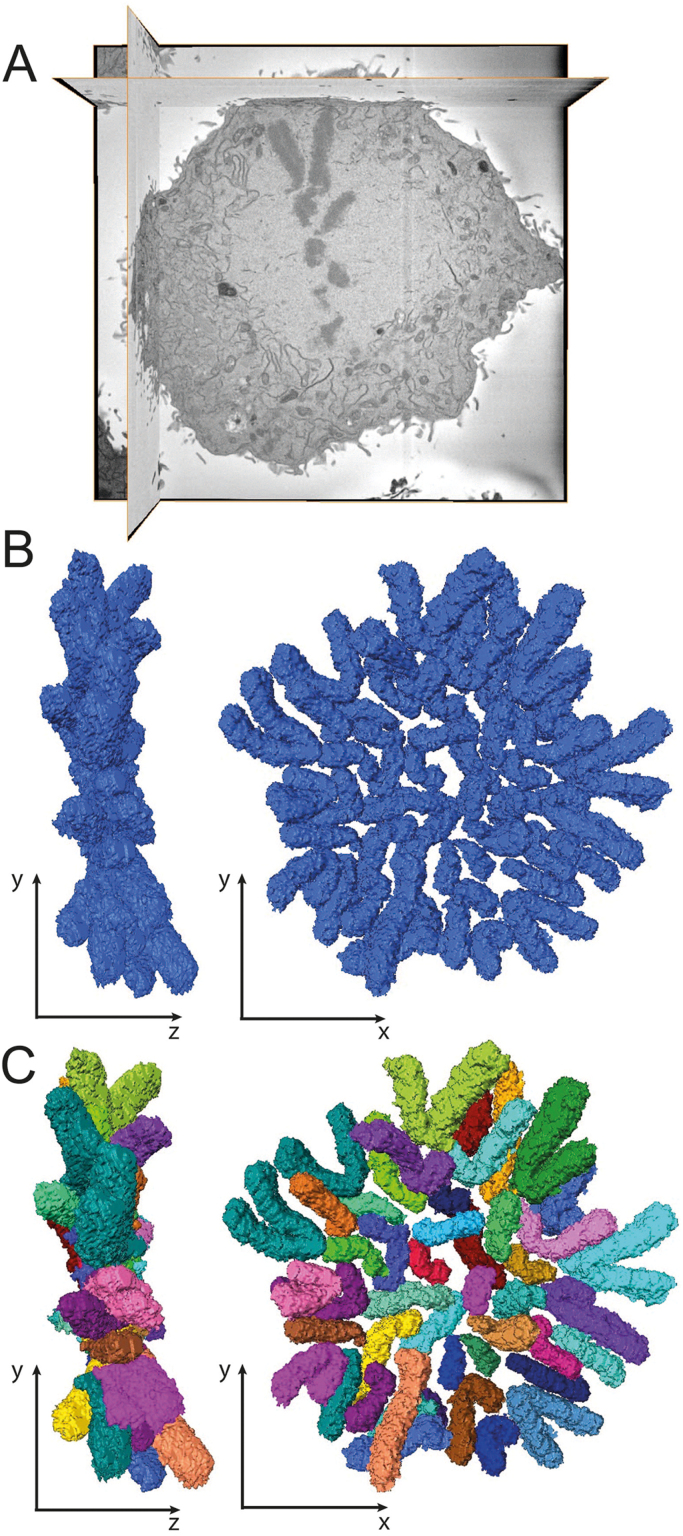
3D-Reconstruction of human metaphase chromosomes obtained by SBF-SEM. (A) Section 84 of 225 of a metaphase hTERT RPE1 cell imaged by Serial Block-Face Scanning Electron Microscopy (SBF-SEM) using 4 nm resolution in XY and 75 nm in Z. The microscope was a Gatan 3View. (B) Modelling and 3D reconstruction of chromosomes. Representative images in XY and ZY are shown. (C) Segmentation and labelling of chromosomes from the 3D reconstruction. 46 chromosomes were resolved and each is shown in a different colour. Chromosomes 1–5 and 19–22 are identified unambiguously by their relative lengths and volumes. Image processing used the program AMIRA (Thermo Scientific).

Advances in microscopy techniques continue to offer new insights into chromosome structure. One example is serial block face scanning electron microscopy, which makes it possible to reconstruct whole mitotic cells at a resolution of 4 nm in X and Y. The resolution in Z depends on the thickness of the sections cut within the microscope, which varies depending on the apparatus used (Fig. 6) [165], [166]. This technique allows, for example, accurate determination of chromosome lengths and volumes, key parameters when assessing models of chromatin fiber packing. A recent study combining an alternative 3D reconstruction strategy (focused ion beam/scanning electron microscopy, or FIB/SEM) with three dimensional-structured illumination microscopy (3D-SIM) has reported, paradoxically, that the scaffold appears to be a double helical structure within each sister chromatid [167]. Other recent studies claim that regular bridges that contain scaffold proteins and connect sister chromatids are critical for the process of mitotic chromosome formation [52], [53]. Currently the method that achieves the best resolution at the level of light microscopy appears to be a combination of expansion microscopy with super-resolution STORM imaging [168], [169], [170]. This technology can resolve individual protein complexes in the context of whole mitotic chromosomes (unpublished results). Considering these new developments, it appears likely that technological advances will continue to provide novel insights and fuel new controversies into the exact nature of the chromosome scaffold.

## 6 Identification, localization and functions of scaffold proteins

In early studies, it was observed that histone-depleted chromosomes [105] and isolated chromosome scaffolds [107] had similar patterns of proteins in 1-dimensional SDS-PAGE. About 30 polypeptides were detected, most of them larger than 50 kDa [105], [107] (Fig. 2). However, at that time it was not clear which of these were true structural proteins of chromosomes, which might have non-structural functions, and which were contaminating cytoplasmic “hitchhikers”.

We now know that, if we ignore the kinetochore (which has more than 100 proteins associated with it at one time or another [171], [172], [173], [174], [175], [176], [177], [178], [179]), it takes remarkably few proteins to convert an interphase nucleus into something that looks like mitotic chromosomes [164]. However, determining the protein composition of mitotic chromosomes is far from simple. Chromosomes are highly charged, and after nuclear envelope breakdown many cytoplasmic proteins bind to them and remain tightly associated throughout subsequent subcellular fractionation procedures. Distinguishing *bona fide* chromosomal proteins that have some role in chromosome structure or segregation from the thousands of fortuitous hitchhikers (many of which are very abundant) is far from simple. This problem plagued early proteomic studies of chromosomes [180], [181], [182], [183], [184], and although interesting proteins were identified, a definitive chromosome proteome remained elusive. This problem was partly overcome by the development of multiclassifier combinatorial proteomics (MCCP), an approach based on a random forest machine-learning algorithm that compares the composition of mutant and wild-type chromosomes and mitotic cell fractions [171]. The more recent chromatin enrichment for proteomics (ChEP) method, which identifies proteins that can be crosslinked to DNA prior to subcellular fractionation [185], has provided further insights.

The conclusion from these comprehensive proteomic studies of isolated chromosomes and mitotic cells is that a handful of histones makes up the bulk of the chromosomal protein mass [171], but that the characteristic mitotic chromosome shape is built as a result of the action of the two condensin complexes, the cohesin complex, the chromokinesin KIF4A and DNA topoisomerase IIα. Targeted proteomic studies of isolated chromosome scaffolds have not yielded any other candidates for major structural proteins [171], [186], [187], [188]. Another as-yet incompletely defined set of highly abundant proteins, largely from nucleoli, makes up the mitotic chromosome periphery compartment (MCPC), which comprises roughly 40% of the mitotic chromosome volume [165], [186], [189], [190]. The remainder of the chromosome mass is composed of thousands of less abundant hitchhiker proteins that associate with the mitotic chromosomes, but likely do not have roles in either chromosome structure or segregation [171], [186], [188].

### 6.1 DNA topoisomerase Iiα

The first step toward identifying the structural proteins of the scaffold was made by Catherine Lewis in the Laemmli lab, using newly developed isolation procedures that yielded chromosomes relatively free of cytoskeletal proteins and other contaminants. Using these chromosomes, Lewis and Laemmli identified two major high molecular weight proteins in the scaffold which they called Sc1 (170 kDa) and Sc2 (135 kDa) [191].

The first of these to be identified was Sc1, which was found to be DNA topoisomerase IIα (topo IIα) [125], [127], [128], [192]. Ever since isolation of the first topo II mutants in *S. pombe* it has been known that topo II activity is required for sister chromatid separation during anaphase [193], [194], [195], but its roles earlier in mitosis remain unclear. A variety of approaches, including indirect immunofluorescence, immunoelectron microscopy and in situ immuno-crosslinking, showed that topo IIα was localized to the axes of sister chromatids [91], [125], [128], [196], [197]. Later studies on the behaviour of *Drosophila* topo II in live embryos [198], and experiments employing over-expression of GFP-tagged exogenous topo IIα in cultured cells (and based on rapid turnover in FRAP experiments) [199], questioned this axial localization and the scaffold role of topo IIα [200]. However, more recent experiments using knock-in technology to tag the endogenous protein confirm the original axial localization ([135], Kumiko Samejima, unpublished). Although a number of experiments have demonstrated that topo IIα is required for assembly and maintenance of mitotic chromosomes [135], [201], [202], [203], it is still not clear whether it plays a structural or only a catalytic role.

In *Drosophila*, depletion of topo II causes dramatic chromosomal structural abnormalities [195]. By contrast, most studies in vertebrates have found that topo IIα inhibition with bis(2,6- dioxopiperazine) derivatives (which do not cause the accumulation of DNA breaks) [194], [204], [205] or depletion [133], [206] lead to formation of long and thin chromosomes that are otherwise fairly normal morphologically, a phenotype reminiscent of that seen following condensin II depletion [207], [208], [209]. In one recent study using acute depletion of topo IIα with an auxin degron, it was found that topo IIα appears not to be required during prophase for chromosome formation, but that it is required for normal chromosome compaction in prometaphase after nuclear envelope breakdown [135]. Polymer simulations reveal that in order for loop extrusion to form rod-like arrays of loops resembling mitotic chromosomes (see Section 7), it is necessary to postulate the existence of a topoisomerase-like molecule that can pass DNA strands through one another, thereby preventing the formation of knots and tangles as the DNA is twisted [90]. Indeed, this may be one reason why topo IIα is associated with the chromatid axis near the bases of the loops [125].

Possible additional role(s) of topo IIα in mitotic chromosome formation are still under investigation. Surprisingly, a recent study reported that degradation of topo IIα during prometaphase and metaphase results in a loss of normal chromosome architecture [135], analogous to that seen many years ago when condensin subunits were depleted from *Xenopus* egg extracts [130] (see next section). The role of topo IIα in maintaining mitotic chromosome structure is unknown, though it could be required to prevent tangles due to ongoing loop-extrusion by condensins [210].

### 6.2 SMC proteins and condensin

**Fig. 7:**
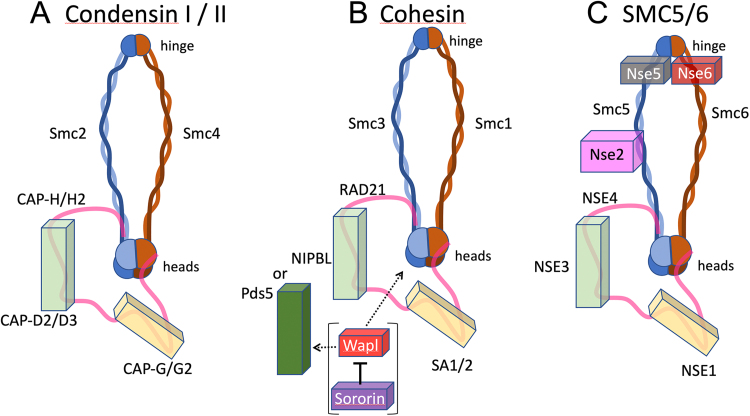
Organization and protein composition of the three SMC protein complexes. Nomenclature is for the vertebrate proteins. For a description of the nomenclature in various model organisms, see [147].

Scaffold protein Sc2 was identified as SMC2 by expression vector cloning using antibodies to the protein from chicken chromosome scaffolds [129]. At the same time the SMC protein family was described in a number of studies of chromosomal proteins from budding and fission yeasts, chicken, *X. laevis* and *C. elegans* [129], [130], [211], [212], [213], [214]. SMC2 was shown to be a component of the condensin complex identified by Hirano and coworkers [63], [130], [131], [215], [216]. Indeed, SMC proteins make up the heart of the condensin and cohesin complexes, both of which play important roles in chromosome organization. They also help make up the SMC 5/6 complex, whose function is still debated [147], [161], [217]. The composition and organization of these three SMC complexes is presented in Fig. 7.

The original SMC mutants were identified as necessary for the segregation of plasmid minichromosomes in budding yeast [218]. SMC initially stood for “Stability of Mini Chromosomes” but was soon rebranded as “Structural Maintenance of Chromosomes”. SMC proteins are also present in prokaryotes, where they are essential for chromosomal structure and segregation [63], [159], [219], [220].

The identification of condensin was a key advance. Working in the Mitchison lab, Tatsuya Hirano identified two proteins XCAP-C and XCAP-E (later identified as SMC4 and SMC2, respectively) as being required for the structure and stability of mitotic chromosomes assembled from sperm nuclei in *Xenopus* egg M-phase extracts [130]. Subsequent immuno-fractionation of those extracts yielded a large complex with SMC2, SMC4 and three other subunits that was essential for the transformation of added nuclei into mitotic chromosomes [131]. This complex was named condensin. Early functional studies showed that yeast SMC dimers lacking the auxiliary subunits could drive DNA annealing independently of ATP hydrolysis [221] and that *Xenopus* condensin could cause ATP-dependent supercoiling of DNA plasmids [222], [223]. Whether or how these activities contribute to mitotic chromosome formation is still not known.

Hirano and co-workers subsequently discovered that many eukaryotes have two such complexes, condensin I and condensin II, with distinct but overlapping functions [207], [208], [224]. Condensin II is nuclear in vertebrates, whereas condensin I is mostly cytoplasmic during interphase [224], [225]. Both condensins localize to the chromatid axes during mitosis. A third variant form of condensin complex is responsible for regulating dosage compensation in *C. elegans* [214], [226], [227], [228].

Early RNAi studies showed that condensin was required for proper chromosomal localization and function of topo IIα [229], [230]. Condensin was also found to be required for the segregation of the repetitive yeast rDNA locus [231], likely dependent on topo II activity. Genetic analysis in *Drosophila* has revealed that condensins are required for sister chromatid resolution and segregation in mitosis [230], [232], [233].

**Fig. 8:**
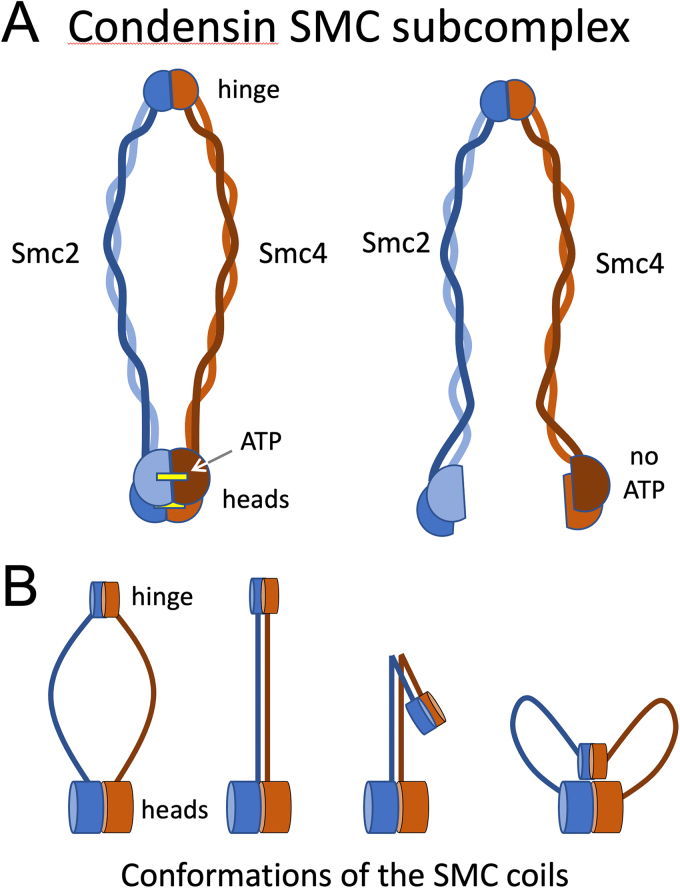
Structural dynamics of SMC complexes. (A) ATP binding regulates the pairing of SMC protein heads. The heads pair, closing the SMC ring, when ATP is bound, and open following ATP hydrolysis. The strap-like kleisin subunit which also joins the two heads together, is not shown in this diagram. (B) The coiled-coils of SMC proteins are highly dynamic. They can form either a ring [267], [461], a closed I-structure [235], [236], [237], [461], [462], a folded-over I-structure [238], [239] or a butterfly structure [463]. The distribution of these different states appears to be regulated by ATP-binding [239], [463]. Since both the hinge region and the HEAT repeat subunits can bind DNA, it is highly likely that these conformational changes contribute to loop extrusion, although the detailed mechanism is still under investigation.

Initial bioinformatic analysis [129], [234] revealed that SMC proteins are related to ABC-ATPases, with Walker A and B sites at opposite ends of the molecules separated by a lengthy region predicted to form two coiled-coils flanking a globular hinge region. The Walker A motif is required for ATP binding whereas the Walker B motif is required for ATP hydrolysis. It was proposed that the molecule might fold back on itself like a jackknife, bringing the Walker A and B sites together [234]. Although initially disregarded, this model gained support from single-particle electron microscopy [235], and was subsequently confirmed by cross-linking mass spectrometry [236] and more recently by molecular structures from x-ray diffraction [237] and cryoelectron microscopy [238], [239]. As in other ABC-ATPases, ATP binding causes the Walker A and B sites to dimerize, driven by the so-called signature motif (another conserved feature of ABC ATPases and SMC proteins). ATP hydrolysis releases them from each other [240], [241]. In condensin, ATP binding causes the Walker A site of SMC2 to dock with the Walker B site of SMC4 and vice versa (Fig. 8). (The corresponding polypeptides are SMC3 and SMC1 in cohesin. For a discussion of the evolution of the ATPase sites in SMC proteins see [242].) Recent studies reveal that the two ATP binding pockets are not functionally equivalent [243]. Since both the hinge region and heads of SMC2 and SMC4 associate with one another, ATP-binding and hydrolysis provides a mechanism regulating the opening and closing of a ring approximately 40 nm in diameter formed by the long coiled coils.

Condensins I and II share the same SMC core (a dimer of SMC2/SMC4), but two different sets of auxiliary subunits (Fig. 7A). The auxiliary subunits include a strap-like kleisin (from the Greek word for closure: kleisimo) that links the ATPase heads of SMC2 and SMC4, stabilizing the closed ring conformation, and two HEAT-repeat-containing subunits (HAWKs, for HEAT proteins Associated With Kleisins) with DNA-binding activity [147], [156], [157], [161], [244], [245], [246], [247], [248]. The kleisin subunits are CAP-H in condensin I and CAP-H2 in condensin II. The HEAT-repeat subunits are CAP-D2 and CAP-G in condensin I, and CAP-D3 and CAP-G2 in condensin II. Despite their obvious similarities, Condensin I and II have significant differences. Condensin II is nuclear during interphase, whereas condensin I is cytoplasmic [224], [225]. Condensin I, but not condensin II, requires an association with the chromokinesin KIF4A to localize to the mitotic chromosome axis [249]. Condensin II, but not condensin I, requires an apparently non-catalytic activity of protein phosphatase 2A to associate with chromosomes in mitosis [250], [251].

**Fig. 9:**
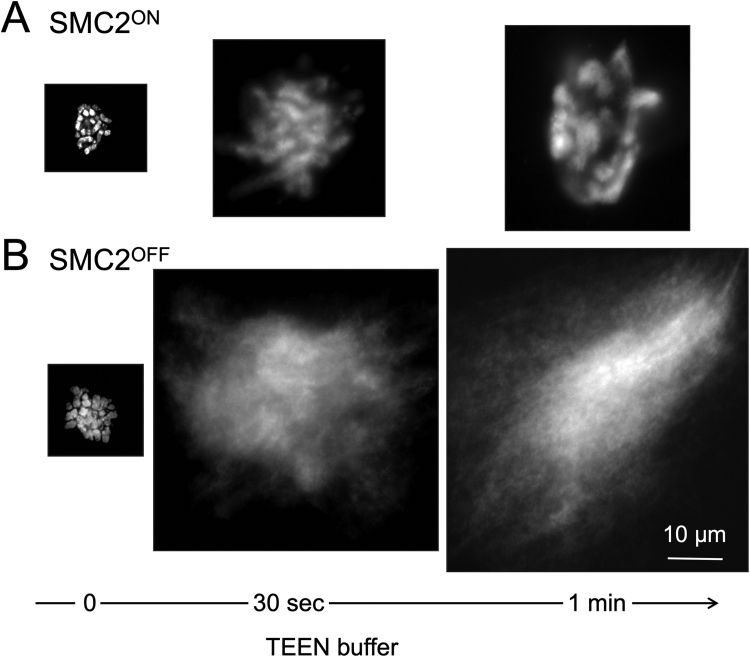
Condensin is required to establish a stable mitotic chromosome architecture. Chromosomes in mitotic chicken DT40 cells either containing SMC2 (A) or depleted of SMC2 and therefore condensin (B) were placed in low ionic strength TEEN buffer in the absence of divalent cations [87]. (A) Chromosomes in cells containing SMC2 expand, but retain a recognizable chromosome morphology. (B) Chromosomes in cells depleted of condensin look relatively normal initially though slightly swollen (left-hand micrograph), but after addition of TEEN buffer, they unravel completely. Experiment performed by Paola Vagnarelli [132].

Condensins shape the mitotic chromosomes, apparently by acting in a pathway with topo IIα [132], [133], [142], [210], [229], [230], [252], [253], [254], [255], [256], [257], [258], [259], [260]. They are thought to do this by forming chromatin loops by loop extrusion as discussed in detail in Section 7. Gradual depletion of either condensin complex by RNAi or conditional gene knockouts does not completely abolish mitotic chromosome architecture [132], [207], [208], [261], [262], although the resulting chromosomes are more fragile than wild type (Fig. 9) [87], [132], [162]. In contrast, depletion of condensin from *Xenopus* egg extracts [131] or acute ablation of condensin either with a degron or by protease cleavage blocks the formation of recognizable mitotic chromosomes [134], [263].

### 6.3 Cohesin

The second major chromosomal SMC complex is cohesin [264], [265]. Cohesin got its name because it establishes cohesion between sister chromatids in a process linked with DNA replication. Cohesin can also bind to chromosomes and form loops throughout the cell cycle, but it normally establishes sister chromatid cohesion only during DNA replication [266], [267]. An exception is that cohesin can also be recruited and establish cohesion during G_2_ in association with the repair of double-stranded DNA breaks [268], [269], [270].

In most eukaryotes the bulk of cohesin is released from chromosomes during the process of condensation via a prophase pathway [271], [272], [273], [274], [275], leaving a peri-centromeric sub-population that is responsible for maintaining sister chromatid cohesion until the kleisin subunit is cleaved by the protease separase to trigger sister separation at anaphase [61], [276], [277], [278], [279]. Cohesin is not believed to be essential for assembly of the chromosome scaffold [280] although recent studies reveal that it does influence the process of chromosome formation (unpublished data).

Like condensin, cohesin is built around a dimer of SMC proteins, SMC1 and SMC3, complexed with three other subunits (Fig. 7B). The following uses the nomenclature for human cells. For the corresponding names in various model systems, see [147]. The SMC-associated subunits include the kleisin Rad21 [281]; the HAWKs SA1 or SA2 [282] and either the cohesin-loader NIPBL [283], [284], [285] or the regulatory subunit Pds5 (both of which are also HAWKs) [271], [286], [287]. NIPBL and Pds5 compete for the same binding site on Rad21. Pds5 can stabilize cohesin’s association with DNA by recruiting the acetyltransferase Esco1/2 [288], which acetylates SMC3 during S phase in a process coupled to DNA replication [289], [290] and recruits (in metazoans only) a binding partner, Sororin, that stabilizes cohesin on DNA and is required to establish sister chromatid cohesion [291], [292], [293]. Alternatively, Pds5 can promote the release of cohesin from DNA by recruiting the release factor Wapl [294], [295]. Wapl and Sororin bind to the same site on Pds5 [296], so a competition between these factors apparently regulates the stability of interphase chromatin loops formed by loop extrusion (see Section 7). Cleavage of the kleisin subunit by the protease separase is responsible for the final separation of sister centromeres that marks the onset of anaphase [61], [276], [277], [278], [279].

Importantly, the realization that mutants in the cohesin loading subunit NIPBL cause Cornelia de Lange Syndrome (CdLS), a developmental disorder resulting in severe malformations and mental retardation [297], [298], [299], led to the discovery of a class of human genetic diseases known as cohesinopathies [300], [301], [302]. CdLS and Roberts Syndrome are the best known of these diseases [303], [304]. It is now known that mutations in at least 4 cohesin-associated genes are associated with CdLS [305], [306]. Roberts Syndrome is caused by a defect in the acetyltransferase Esco2 [307], which acetylates SMC3 to establish stable sister-chromatid cohesion [308]. Analysis of the phenotypes of cohesin mutants has revealed that the complex plays an important role in organizing the interphase nucleus and regulating patterns of gene expression. The cohesin complex acts with the barrier factor CTCF to organize interphase chromosomes into local loop domains of 500 kb – 1 Mb known as TADs (“topologically associating domains”) [309], [310], [311]. TADs may, at least in some instances, regulate the access of enhancers to their target genes, and thereby help regulate gene expression. TADs may correspond to the bands in insect polytene chromosomes [312], one of the oldest observations supporting an organized substructure for interphase chromosomes [313]. The interphase functions of cohesin are beyond the scope of this chapter but are discussed in recent reviews [157], [314].

### 6.4 SMC 5/6 complex

The function of a third SMC complex has proven to be more elusive, so this complex is known only by the name of its SMC proteins: the SMC5/6 complex. This is the most elaborate of the three eukaryotic SMC complexes, with 4–6 non-SMC subunits (Fig. 7C). Unlike condensin or cohesin, SMC5/6 complexes possess associated ubiquitin and SUMO ligase (E3) activity conferred by three of the non-SMC subunits. The complex can associate with unusual DNA structures (e.g. ssDNA, RNA/DNA hybrids, supercoils or catenanes) and compact the surrounding DNA [315], [316].

SMC5/6 association with DNA is unchanged as cells progress from G_2_ into mitosis (Itaru Samejima, unpublished). The complex has been reported to concentrate near centromeres and the rDNA, though different localization has been seen in different studies. Depletion of SMC5 has no effect on the chromosomal association of condensins or cohesin [186]. The SMC5/6 complex appears to function during S phase in replicating and separating regions of repetitive DNA and may also have roles in DNA repair and recombination. Depletion of SMC5/6 components prior to S phase entry causes dramatic chromosome segregation defects in mitosis, apparently reflecting difficulties with resolving topological tangles [317].

### 6.5 Chromokinesin KIF4A

The least explored component of the chromosome scaffold is the chromokinesin KIF4A [133], [318], [319], [320], [321], [322]. Chromokinesins are a subgroup of the kinesin family of motor proteins that bind both to DNA [152] and to microtubules. KIF4A is a bona fide kinesin that can move cargoes to microtubule plus ends in neurons [323] and has a key role in forming the microtubule structures of the central spindle and intercellular bridge as cells divide [324], [325], [326], [327]. KIF4A is unusual for a kinesin, in that it has also been found to be required for DNA damage responses [328], [329] and decreased KIF4A activity is associated with cancer [330], [331], [332] (reviewed in [333]). The underlying mechanisms are not known.

KIF4A works together with condensins and topo IIα to shape mitotic chromosomes [133], [249], [320], [321], [333], [334]. As a chromokinesin, KIF4A can bind both to DNA [152], [335], [336] and to condensin [133], [249], [320], [334], [335], [337] and it is required for normal condensin I localization on chromosome axes [133], [249]. Indeed, KIF4A depletion reduces condensin I levels on isolated chromosomes, while levels of condensin II and the other SMC protein complexes are not affected [133]. Depletion of KIF4A leads to production of short, fat chromosomes similar to what is seen with loss of condensin I [133], [207], [208], [334] and chromosomes formed in its absence have a defective structure as shown in the intrinsic metaphase structure (IMS) assay [132], [133]. Because KIF4A functions as a dimer and has a DNA-binding site [152], it is possible that it could contribute to the formation or stabilization of DNA loops, although there is as yet no direct experimental evidence for this. At this point, the mechanism of KIF4A action during mitotic chromosome formation remains unclear.

### 6.6 Interactions between scaffold proteins

These five proteins – topoisomerase II, condensins I and II, cohesin and the KIF4A chromokinesin – constitute the minimal requirements for reconstitution of condensed chromosomes in vitro [164], [255], [338]. Thus, they appear to be the major, if not the only, essential components of the chromosome scaffold. Despite the small number of major proteins involved, the analysis of chromosome condensation is complicated by the fact that the activity of all of these proteins is regulated by interactions between them, by phosphorylation [187], [322] and by changes in compartmentalization during chromosome formation [207], [225].

Condensin interacts functionally and structurally with topo IIα. Experiments in budding yeast reveal that condensin-mediated chromatin supercoiling may promote topoisomerase II activity [253]. In addition, lowering condensin levels by only 40% is sufficient to disrupt the concentration of topo IIα along the chromatid axes [134]. Indeed, simultaneous RNAi experiments showed that topoisomerase II, condensins and KIF4A somehow counterbalance one another during mitotic chromosome formation. Morphological disruptions caused by condensin and KIF4A depletion were to some extent rescued if topo IIα was depleted at the same time [133].

Topo II activity is regulated by a number of different posttranslational modifications. For example, the enzyme is ADP-ribosylated [339], and this can inhibit its activity [340]. Topo II is phosphorylated during mitosis [180], [341], [342], [343], [344], [345], [346], [347], and this has generally been shown to stimulate its activity [348], [349], [350], [351], [352], possibly by alleviating auto-inhibition by the C-terminal tail of the protein [346], [351], [353], [354], [355], [356]. Topo II SUMOylation regulates the spindle assembly checkpoint via Haspin and Aurora B kinases [357], [358].

Condensin phosphorylation has been reported to be required for its activity in condensing chromosomes [359], [360], and a number of kinases have been reported to be involved. Aurora B kinase is important for the correct localization and function of condensin on mitotic chromosomes [224], [320], [361], [362], [363], [364], [365]. Aurora B phosphorylation has been proposed to regulate condensin association with H2A and/or H2A.Z, which can act as chromosomal receptors for the complex [366]. Phosphorylation of CAP-H2 by the kinases Mps 1 and Plk1 is important for condensin II binding and chromosome condensation [367], [368]. In contrast, phosphorylation by Chk2 kinase after DNA damage inhibits condensin association with DNA [369]. Phosphorylation by casein kinase II during interphase has also been reported to inhibit condensin I activity [370], and phosphorylation of the SMC hinge of *S. pombe* condensin reduces its binding to DNA [371].

Although the role of condensin’s plasmid supercoiling activity [222] in mitotic chromosome condensation remains uncertain, a number of studies have looked at the regulation of this activity by phosphorylation. Phosphorylation of CAP-D2 or SMC4 was reported to stimulate condensin DNA supercoiling activity [359], [372], [373]. A similar effect was seen following phosphorylation of the three non-SMC subunits by yeast polo kinase [374].

KIF4A phosphorylation is required for its association with condensin I [249], [375] and for lateral chromosome compaction in mitosis [375]. Since condensin I and KIF4A appear to be mutually interdependent for their association with chromosomes, this could possibly explain why KIF4A association with chromosomes requires phosphorylation [337]. Aurora A promotes chromosome congression by activating the condensin-dependent chromosomal pool of KIF4A [321]. During later stages of mitosis, KIF4A not only has important functions in mitotic chromosome formation, it also has important functions in the mitotic spindle, where a balance between Aurora B and PP2A-B56 activity acting on KIF4A appears to inhibit microtubule dynamics in the central mitotic spindle [376], [377], [378].

## 7 Formation and stabilization of the chromatin fiber loops in mitotic chromosomes

Contemporary notions of mitotic chromosome organization favor “bottle-brush” models with radial loops as proposed by Laemmli and coworkers [21], [44], [108]. It now appears that SMC complexes are responsible for this loop organization in both interphase and mitosis.

The discovery of an ATP binding site in the N-terminal domain of cohesin subunit SMC1 led to the proposal that SMC proteins might be molecular motors that could function by looping DNA [211]. This fueled speculation that SMC complexes might form loops by an active extrusion process similar to “DNA reeling” [379] in which the ring-like SMC complex would bind to a site on the DNA and then use its motor activity to “slide” away from this, effectively pulling the DNA backwards and extruding it as a loop through the SMC ring [380]. Subsequent work supports the notion of loop extrusion by condensin [381], [382], [383], [384], [385] and by cohesin [386], [387] (reviewed in [384], [388], [389]).

Indeed, isolated yeast condensin has been shown to have motor activity and move along DNA [390], and in elegant single-molecule studies, condensin has been shown to actively extrude loops of DNA in an ATP-dependent fashion [385], [391], [392], [393]. It proved more challenging to find conditions where cohesin would actively extrude loops in vitro, but this has since been achieved following the discovery that the activity depends on the presence of the cohesin loader NIPBL [386], [387]. Recent estimates are that the loops made by condensin and cohesin in vitro average 100–200 kb in size, produced by complexes that have a lifetime on the DNA of 5–30 min [389].

An important question is whether these loops tend to form at particular DNA sequences or whether the SMC binding sites are more promiscuous. Here, there are clear differences between the situations in mitosis and interphase. Older studies that sought to find mitotic chromosome scaffold associated regions (SARs) and the analogous nuclear matrix associated regions (MARs) initially ended in frustration. They were based on isolating nuclear matrix/chromosome scaffold fractions following restriction endonuclease digestion and looking for the enrichment of particular sequences. The results were difficult to interpret.

For nuclei, some MARs were mapped, especially in relation to transcriptionally active loci (e.g., [394], [395], [396], [397]. Evidence has variously been obtained for preferential association of the nuclear matrix with supercoiled DNA, bent DNA, single-stranded DNA, and, in cases of some particular genes, specific DNA sequences [398], [399], [400]. The functional significance of these sequences remains obscure. Instead, it has turned out that many interphase chromatin loops have convergent sites for the architectural factor CTCF (CCCTC binding factor) at their base. CTCF can interact with cohesin, and loop-extruding cohesin is found together with CTCF at the base of interphase chromatin loops [401], [402], [403], [404]. Thus, there are two discrete populations of cohesin: cohesive cohesin and loop-extruding cohesin. Cohesive cohesin tends to accumulate at the ends of genes, where it is presumably pushed by the transcriptional machinery [405], [406], [407]. Since CTCF binds to sites containing the sequence CCCTC, there is indeed a sequence preference (albeit degenerate) at the base of interphase chromatin loops [309], [408], [409]. In the absence of CTCF or cohesin, the TAD/loop organization of interphase chromatin is lost [410].

For mitotic chromosomes, attempts to identify particular SARs were not successful, although it was determined that some scaffold proteins bind preferentially to DNA with runs of A:T where the minor groove is narrower [196], [411], [412], [413], [414], [415]. It was possible that the failure to find specific SARs might be because scaffold protein positions on the DNA were randomized during the extraction process required for scaffold isolation. However, subsequent ChIP-seq mapping of condensin revealed very little association with specific DNA sequences [416], [417]. Although some preferential localization was found near centromeres, telomeres and at some promoters, the bulk of condensin appears to be distributed essentially randomly across mitotic chromosomes. Thus, attempts to identify a consensus SAR motif were doomed to failure. With the loop extrusion mechanism there is no reason to require that specific DNA sequences be associated with the scaffold, though there could still be a bias toward certain sequences or types of DNA (e.g., AT-rich, supercoiled, etc.). The idea of loop extrusion also makes sense evolutionarily as it means that the general shape of the chromosome will not be affected even by gross changes in DNA sequence such as the addition or removal of large amounts of repetitive DNA, or large-scale chromosomal rearrangements.

Polymer modelling has subsequently revealed that loop extrusion on its own can compact the DNA [381], [383], [418]. Furthermore, given a dense bottle-brush-like array of loops, entropic forces resulting from molecular volume exclusion effects between adjacent loops will inevitably cause the compacted polymer to adopt a rod-like shape similar to that produced during mitotic chromosome formation [419]. Thus, loop formation driven by DNA extrusion will result in the production of rod-shaped chromatids without the need for a scaffold superstructure to actively shape the chromosome [383]. At time of writing, loop-extrusion models are clearly in the ascendency; however, they are not universally accepted. An alternative “diffusion capture” model [420], based on the ability of cohesin to link sister chromatids by trapping the replicated DNA molecules within its coiled-coil loop, proposes that SMC complexes can sequentially entrap two DNAs that are brought into proximity by Brownian motion [421]. A problem with such diffusion capture models is that they do not explain how loop formation occurs strictly *in cis* on a single DNA molecule rather than allowing condensin to “capture” DNA from sister chromatids or even different chromosomes. During mitotic chromosome formation loop extrusion clearly works by acting only *in cis*. However, the diffusion capture models cannot be excluded at present.

## 8 Genomic approaches to study chromosome structure

Studies of chromatin organization in cell nuclei and chromosomes were revolutionized in 2002 when Job Dekker developed the 3C (“Chromatin Conformation Capture) method [422]. The basic idea of “C” approaches is to crosslink the DNA in intact nuclei or chromosomes using formaldehyde, isolate the DNA, fragment it with nucleases, ligate it under dilute conditions so that only crosslinked segments will be joined end-to-end, reverse the crosslinking, and analyze the resulting DNA chimeras by PCR (3C, 4C - [422], [423]) or deep sequencing (Hi-C [424]) (reviewed in [310], [425], [426]). Many other variations on this approach are now in use as well.

In this type of analysis, the ligation of two distant sequences shows that they were close together in the nuclei or chromosomes. Hi-C data are often presented in the form of a two-dimensional “heat map” in which the color indicates the frequency of association of two sequences, a measure of their spatial proximity. For mathematical analysis, the data are displayed as contact probability plots in which the probability of two sequences being ligated together is plotted as a function of their distance apart on the chromosome. Hi-C is powerful, but expensive. In the initial study, achieving a resolution of 1 Mb required 10^7^ sequence reads [424], with roughly 100-fold more reads being required for every 10-fold gain in resolution [425].

These methods have provided important information for mapping nuclear organization and have helped to elucidate aspects of eukaryotic gene expression such as the spatial relationships between transcribed regions and enhancers. Heat maps of Hi-C applied to interphase nuclei reveal complex but consistent features including loops, topologically associated domains (TADs - [309]) and compartments [310], [424], [427], [428], [429]. TADs are generally organized by CTCF and cohesin and consist of local clusters of loops spanning regions of ~1,000,000 bp [311]. TADs are often, though not invariably, linked to gene regulation (e.g., genes may be transcribed more efficiently if their enhancer is located within the same TAD). Compartments are much larger regions. They reflect a tendency of active regions of chromatin to associate with one another (A compartments) and inactive regions to associate with one another (B compartments) (references above). This may possibly be a result of liquid-liquid phase separation [28].

## 9 Hi-C studies of mitotic chromosome structure and formation

In contrast to results obtained with interphase nuclei, the application of Hi-C to HeLa mitotic chromosomes showed a much more homogeneous heat map [90]. The TADs and compartments characteristic of interphase nuclei disappeared [430], and a relatively simple pattern was observed which was essentially the same for all chromosomes and for all loci within a chromosome. Naumova et al. [90] concluded, using polymer simulations, that their data for mitotic chromosomes were best explained by a linear array of chromatin loops arranged consecutively along the length of the chromatid. Their data would be inconsistent with models involving hierarchical levels of coiling.

A recent study of mitotic chromosome formation in cultures undergoing synchronous mitotic entry has reconciled apparently contradictory aspects of several previous models of mitotic chromosome organization [89]. This study was made possible by the development of a chicken DT40 cell line in which chemical genetics [431] was used to place mitotic entry under the control of a modified *Xenopus* Cdk1^as^ (analog-sensitive) kinase whose activity could be regulated by the ATP analogue 1NM-PP1 [432], [433], [434]. When these cells are incubated in 1NM-PP1, they accumulate at a point in very late G_2_ phase. Upon wash-out of the drug, they enter mitosis within minutes with a high degree of synchrony. For this study, the CDK1^as^ allele was combined with auxin-mediated acute ablation [435] of condensin I or II to yield a time-resolved genetic analysis of mitotic chromosome formation.

Analysis of Hi-C contact probability curves and subsequent modeling by Gibcus et al. [89] revealed that all long-range interactions that existed between chromosomes during interphase were rapidly lost shortly after release from the 1NM-PP1 block. Compartments disappeared within 5 min (corresponding to early prophase), and this was followed shortly thereafter by a loss of TADs. Analysis of the Hi-C data revealed that these “granular” chromatin interactions are replaced by an organization in which the entire chromosome consists of sequence-independent randomly positioned loops of about 60 kb. Because condensin II is stably associated with the chromosome, these loops continue to grow as mitosis progresses. Morphologically, the looped structures detected by Hi-C at this stage corresponded to prophase chromosomes. The formation of the loops was most likely due to loop extrusion by condensin II, which is nuclear in interphase [224], [225]. However, some contributions from cohesin could not be ruled out.

RNAi of condensin II causes a delay in chromosome condensation, causing it to begin only in late prophase [89], [225]. Condensin I is generally cytoplasmic until Nuclear Envelope Breakdown (NEB), but it is likely that the late prophase condensation seen in the absence of condensin II is a result of cytoplasmic condensin I entering the nucleus following the loss of barrier function by the nuclear pores. This occurs several minutes before visible NEB [436], [437], [438].

A dramatic transition was seen in the Hi-C data at Nuclear Envelope Breakdown (NEB), which defines the transition from prophase to prometaphase. A strong second diagonal line appeared in heat maps [89]. This diagonal revealed that all chromosome loci were interacting in a non-sequence-specific way with other loci roughly 2 Mb away in an axial direction along the chromatid. As prometaphase progressed towards metaphase, this second diagonal broadened and moved to larger spatial separations, so that by late prometaphase, it appeared that all chromosomal loci were interacting with other loci roughly 10 Mb away. This agreed with the previous ChIP mapping of condensin in these cells, which showed that most condensin was randomly positioned across the genome [416].

Analysis of the Hi-C contact probability plots revealed that during the transition into prometaphase, the initial 60 kb prophase chromatin loops had grown to an average length of 400 kb, presumably because condensin II continues to extrude the loops. The persistence and growth of these loops is a consequence of the fact that condensin II association with DNA is highly stable during mitosis [262].

**Fig. 10:**
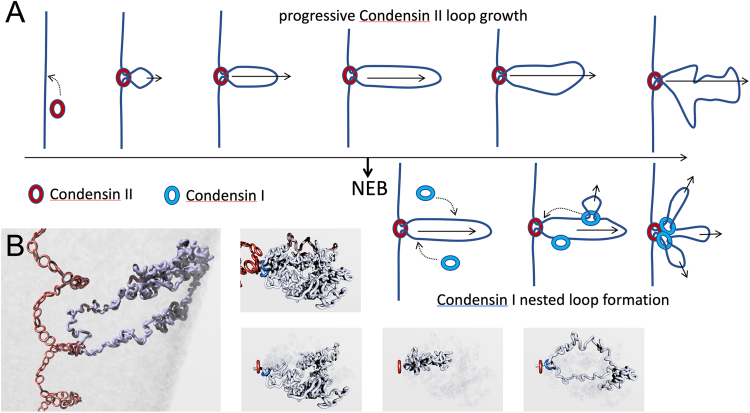
Nested loop extrusion by Condensin I and II. (A) Condensin II begins loop extrusion during prophase, so that by late prophase the average loop size is about 60 kb. By metaphase, the loop size has grown to around 400 kb, but the observed loops are smaller because after nuclear envelope breakdown (NEB) condensin I jumps onto the DNA within the condensin II loops and begins to extrude its own loops [89]. This process depends on the fact that the association of condensin II with DNA is substantially more stable than that of condensin I [262]. (B) Frames from an animation describing mitotic chromosome organization made by Anton Goloborodko [89].

The chromosomes start to come into contact with condensin I from the cytoplasm several minutes prior to NEB. It is presumably this condensin I that drives late prophase chromosome condensation following knockdown of condensin II [225]. Upon NEB, chromosomes are flooded with condensin I, which in DT40 and HeLa cells is present at roughly 5–6-fold higher levels than condensin II [189], [439] and is highly dynamic [262]. Because the chromosomes already consist of linear arrays of chromatin loops formed by condensin II during prophase, condensin I is likely to bind within those condensin II loops, thereby forming nested loops by an extrusion process. Thus, by late prometaphase/metaphase the chromosome has a complex loop structure with 80 kb condensin I loops nested within 400 kb condensin II loops (Fig. 10). Subsequently, this nested loop organization deduced from Hi-C data was independently shown to be consistent with the amounts and dynamics of the condensin I and II subunits in HeLa cells [439].

The kinetics and dynamics of loop extrusion by the two condensins can explain why condensin II-depleted chromosomes are long, thin and floppy, as they have only the shorter, more dynamic condensin I-mediated loops [207], [208]. By contrast, condensin I-depleted chromosomes are shorter and fatter, because the 400 kb loops formed by condensin II are not further compacted via the formation of shorter loops nested within them.

Polymer modeling revealed that the second diagonal on heat maps is best explained by a sequential helical arrangement of loops in which the chromatid axis resembles a helical staircase-like structure, with a loop projecting outward from each step [89]. Thus the 2–10 Mb spacing of the second diagonal likely reflects interactions along the long axis of the chromosome between loops extending from adjacent gyres of the scaffold helix. Genetic analysis indicated that the “ spiral staircase” requires condensin II for its formation, since ablation of condensin II subunit CAP-H2 causes a loss of the second diagonal in Hi-C analysis. The simplest explanation for this is that condensin II molecules assemble in a helical array, likely discontinuous, along the chromatid axis. The fact that interacting loci in prometaphase chromosomes are about 10 Mb apart and condensin II forms 400 kb loops [89] suggests that there are about 25 loops per turn of the condensin II helical staircase.

These experiments were particularly satisfying because they yielded a model that is consistent with all of the previous observations of mitotic chromosome organization: mitotic chromosomes appear to contain a helix of loops of the size measured by Laemmli and co-workers [21], [44], [87], arranged by association with a helical scaffold [91]. These chromosomes would, if examined by traditional electron microscopy, look like a randomly folded fiber or polymer melt. However, in the world of mitotic chromosomes, it is dangerous to become complacent. A recent study used highly detailed three-dimensional modelling from light microscopy to conclude that although short segments of mitotic chromosomes might appear helical, in fact adjacent segments have opposite handedness (known as “perversions”), so that the overall structure is a zig-zag rather than a helix [52]. This study pushed the interpretation of light microscope images to its very limits of resolution, and the jury is still out as to whether chromosomes are indeed perverted structures. However, preliminary polymer calculations suggest that a zig-zag structure would be unlikely to explain the second diagonal seen in Hi-C studies (unpublished results).

## 10 Mitotic chromosome maintenance

One of the greatest unsolved mysteries of mitotic chromosomes has yet to be systematically studied. This is the fact that after chromosomes are formed, they must be actively maintained throughout mitosis. Thus, chromosome morphology does not simply reflect the outcome of an assembly pathway. It also reflects an active process of ongoing maintenance. This was first revealed by Hirano in the study in which he showed that depletion of *Xenopus* XCAP-C and XCAP-E (SMC4 and SMC2) proteins blocked the formation of chromosomes in *Xenopus* egg M-phase extracts [130]. In the same paper, Hirano showed that if XCAP-C (SMC4) was depleted *after* mitotic chromosomes had formed, the chromosome morphology was lost. This aspect of condensin function was not pursued until many years later when it was reinvestigated using acute depletion. If condensin I with a TEV-cleavable kleisin is expressed in *Drosophila* and then cleaved by microinjecting TEV protease, chromosomes rapidly lose their characteristic structure and the DNA becomes highly tangled [210]. In chicken DT40 cells, chromosomes that had assembled a normal mitotic architecture rapidly reverted to a collapsed mass of chromatin following acute depletion of condensin I and II by degron-mediated degradation of SMC2 [134]. Likewise, in fission yeast, inactivation of a temperature-sensitive condensin mutant after mitotic chromosome formation resulted in a failure of chromosome segregation (though the morphology of the chromosomes was not examined in detail) [365]. Thus, condensin is required to maintain ongoing mitotic chromosome structure in vivo as well as in vitro.

The most recent addition to this story comes from the work of Nielsen and Hudson, who showed that what holds true for condensin is also true for topo II. They found that rapid auxin-mediated degradation of topo II in prometaphase also resulted in a loss of the organized mitotic chromosome structure [135]. Previous studies had suggested that acute inhibition of topo II caused a loss of mitotic chromosome architecture [205], [210], [440]. However this could have been due to dominant-negative effects caused by trapping inactivated topo II dimers on the DNA. The Nielsen and Hudson study eliminates that possibility.

These observations call into question the way we have traditionally thought about mitotic chromosomes and may also mean that we need to re-define what we mean by a chromosome scaffold. Rather than being stably assembled structures, mitotic chromosomes can be thought of as ongoing dynamic processes. This is yet more evidence that the chromosome scaffold is not a static structure, like a construction scaffold. Instead, the scaffold can be thought of as a dynamic ongoing association between a set of proteins that form and then actively maintain mitotic chromosome morphology. It is extremely interesting that all of the major scaffold proteins are ATPases, and it may be relevant that in order to function as a scaffold protein KIF4A requires its motor domain [133], [249]. This latter observation has been paradoxical, since microtubules are absent from the nucleoplasm during chromosome formation. It is unlikely that microtubules play a key role in the active maintenance of mitotic chromosome structure since chromosomes are stable in the presence of microtubule depolymerizing drugs.

The process of active mitotic chromosome maintenance remains one of the unsolved (and unstudied) mysteries of mitotic chromosome biology.

## 11 Chromatin compaction during mitosis

As mentioned at the beginning of this chapter, mitotic chromosome formation involves two processes that proceed in parallel. In one, the chromatin is compacted by a relatively subtle factor of 2–3-fold or less. In the other, which has occupied the bulk of this review, the compacted chromatin is shaped into the characteristic rod-shaped chromosomes that gave mitosis its name. This shaping of the chromosomes is driven by non-histone proteins of the chromosome scaffold.

The two processes are separable: compaction of the chromatin can occur even in the absence of condensin and chromosome shaping. As shown originally by Hirano, depletion of condensin subunit SMC4 causes mitotic chromosomes assembled in *Xenopus* extracts to lose their characteristic shape, but the chromatin still remains compact [130]. Acute knockout of condensin prevents discrete chromosomes from forming: instead, the chromatin forms a condensate in which individual chromosomes cannot be seen [134], [263]. This is an area where the techniques used matter greatly. If condensin is knocked out by either RNAi or by shutting off its transcription (so that the protein is lost according to its normal turnover), then morphologically distinct individual chromosomes are still formed [132], [207], [224], [441], [442]. This implies that under those conditions a (slow-acting) alternative pathway is able to form individual chromosomes.

Mitotic chromatin compaction is likely to be important because it facilitates individualization of different chromosomes [443] and separation of sister chromatids [444]. (The latter is now accessible to new Hi-C methods [409], [445], [446].) It will therefore not be surprising if there are multiple redundant mechanisms. For example, it was proposed that chromatin compaction is due to hyperactive condensin DNA supercoiling activity [447], but since this obviously would require condensin it cannot be the whole story. Given the importance of mitotic chromatin compaction, any mechanism arising during evolution that enhanced compaction could have been positively selected. Thus, several distinct mechanisms may contribute, and that makes it a particularly difficult problem to solve.

The cause of the condensin-independent compaction is not known, but several possible mechanisms have been suggested. These range from simple ionic interactions to more complex mechanisms involving histone modifications. Secondary Ion Mass Spectrometry (SIMS) with a scanning ion microprobe revealed that Mg^2+^ binds to chromosomes roughly every 20–30 nucleotides and that Ca^2+^ binding is about twice as frequent [448]. Ca^2+^ appeared to be concentrated near the chromatid axis near topo II, whose activity it can inhibit in vitro [448]. Mg^2+^ was localized more in the peripheral chromatin. Interestingly, images could be obtained where the Ca^2+^ signal appeared to follow a helical path. Others have also proposed that Ca^2+^ can affect chromosome structure and chromatin compaction in vivo [449]. In contrast, it has recently been proposed that a transient rise in free Mg^2+^ due to ATP hydrolysis could contribute to chromatin compaction [450]. ATP has recently been proposed to function not only as an energy source, but also as a biological hydrotrope that helps maintain the solubility of hydrophobic molecules in the cytoplasm [451].

Bradbury and coworkers proposed that chromosome condensation is due to phosphorylation of histone H1 [452], [453]. However, it has since been shown that premature chromosome condensation can be induced in G_1_-phase mammalian cells without histone H1 phosphorylation [454]. *In vitro* reconstitution experiments suggest that core histone posttranslational modifications may be important. When ordered nucleosome arrays are reconstituted in vitro on Widom 601 repeats [455], [456] using purified interphase or mitotic histone octamers with intact post-translational modifications [457], [458], nucleosome arrays assembled from mitotic histones have a greater tendency to aggregate than do those assembled from interphase histone octamers [459]. Importantly, other chromosomal proteins, including condensins, KIF4A and topoisomerase II were absent from these octamer preparations. The combination of mitosis-specific modifications that drive this selective mitotic chromatin compaction remains to be determined.

## 12 Conclusion/perspectives

Since chromosomes were discovered in the 19th century, a tremendous amount has been learned about their formation, composition and structure. Mitotic chromosomes are of intrinsic interest because the dramatic events of mitosis are endlessly fascinating. However, they are also important because deciphering them may help us to better understand the structure and dynamics of other chromosome forms. Knowing the structure of mitotic chromosomes should help us understand interphase, meiotic and polytene chromosomes as well, since all forms of chromosomes are presumably structurally related. It may also yield insights into certain human diseases such as cohesinopathies (e.g., Cornelia de Lange and Roberts Syndromes), as well as the origins and effects of chromosomal abnormalities.

Many challenging questions remain to be addressed. How do the key proteins – condensins I and II, topo II and KIF4A – cooperate to produce two separate, rod-like chromatids as so beautifully seen in the 1960s-era micrographs of Ernest DuPraw? What is the precise mechanism of shaping chromosomes and scaffolds? What is the mechanism of loop extrusion? What are the origins of coiled chromatids, and why are they sometimes seen in isolated chromosomes? We anticipate that these and many as-yet unasked questions will be answered in coming years as new techniques are developed and old ones are applied with new creativity.

## References

[1] Lima-de-Faria A. (2003).

[2] Balbiani E.G. (1861). Recherches sur les phénomènes sexuels des Infusoires. J. Physiol. l’Homme Animaux.

[3] Hertig A.T. (1968). The primary human oocyte: some observations on the fine structure of Balbiani’s vitelline body and the origin of the annulate lamellae. Am. J. Anat..

[4] Hughes A. (1959).

[5] Harris H. (1999).

[6] Paweletz N. (2001). Walther Flemming: pioneer of mitosis research. Nat. Rev. Mol. Cell Biol..

[7] Heuser E. (1884). Beobachtungen über Zellkerntheilung. Bot. Zent..

[8] Waldeyer H.W.G. (1888). Über Karyokinese und ihre Beziehungen zu den Befruchtungsvorgängen. Arch. Mikrosk. Anat. Entwickl..

[9] Sutton W.S. (1902). On the morphology of the chromosome group in Brachystola magna. Biol. Bull..

[10] Gall J.G. (1963). Kinetics of DNAase action on chromosomes. Nature.

[11] Paulson J.R., Harris J.R. (1982). Electron Microscopy of Proteins.

[12] Paulson J.R., Vagnarelli P. (2011).

[13] Llères D., James J., Swift S., Norman D.G., Lamond A.I. (2009). Quantitative analysis of chromatin compaction in living cells using FLIM-FRET. J. Cell Biol..

[14] Martin R.M., Cardoso M.C. (2010). Chromatin condensation modulates access and binding of nuclear proteins. FASEB J..

[15] Vagnarelli P., Earnshaw W.C. (2012). Repo-Man-PP1: a link between chromatin remodelling and nuclear envelope reassembly. Nucleus.

[16] Ou H.D., Phan S., Deerinck T.J., Thor A., Ellisman M.H., O’Shea C.C. (2017). ChromEMT: visualizing 3D chromatin structure and compaction in interphase and mitotic cells. Science.

[17] Kornberg R.D., Lorch Y.L. (1999). Twenty-five years of the nucleosome, fundamental particle of the eukaryote chromosome. Cell.

[18] Thoma F., Koller T., Klug A. (1979). Involvement of histone H1 in the organization of the nucleosome and of the salt-dependent superstructures of chromatin. J. Cell Biol..

[19] DuPraw E.J. (1965). Macromolecular organization of nuclei and chromosomes: a folded fibre model based on whole-mount electron microscopy. Nature.

[20] Davies H.G. (1968). Electron-microscope observations on the organzation of heterochromatin in certain cells. J. Cell Sci..

[21] Marsden M.P.F., Laemmli U.K. (1979). Metaphase chromosome structure: evidence for a radial loop model. Cell.

[22] Langmore J.P., Paulson J.R. (1983). Low angle x-ray diffraction studies of chromatin structure in vivo and in isolated nuclei and metaphase chromosomes. J. Cell Biol..

[23] Paulson J.R., Langmore J.P. (1983). Low angle x-ray diffraction studies of HeLa metaphase chromosomes: effects of histone phosphorylation and chromosome isolation procedure. J. Cell Biol..

[24] Joti Y., Hikima T., Nishino Y., Kamada F., Hihara S., Takata H., Ishikawa T., Maeshima K. (2012). Chromosomes without a 30-nm chromatin fiber. Nucleus.

[25] Maeshima K., Ide S., Babokhov M. (2019). Dynamic chromatin organization without the 30-nm fiber. Curr. Opin. Cell Biol..

[26] König P., Braunfeld M.B., Sedat J.W., Agard D.A. (2007). The three-dimensional structure of in vitro reconstituted Xenopus laevis chromosomes by EM tomography. Chromosoma.

[27] Ricci M.A., Manzo C., García-Parajo M.F., Lakadamyali M., Cosma M.P. (2015). Chromatin fibers are formed by heterogeneous groups of nucleosomes in vivo. Cell.

[28] Gibson B.A., Doolittle L.K., Schneider M.W.G., Jensen L.E., Gamarra N., Henry L., Gerlich D.W., Redding S., Rosen M.K. (2019). Organization of chromatin by intrinsic and regulated phase separation. Cell.

[29] Strickfaden H., Tolsma T.O., Sharma A., Underhill D.A., Hansen J.C., Hendzel M.J. (2020). Condensed chromatin behaves like a solid on the mesoscale in vitro and in living cells. Cell.

[30] Matsuda A., Shao L., Boulanger J., Kervrann C., Carlton P.M., Kner P., Agard D., Sedat J.W. (2010). Condensed mitotic chromosome structure at nanometer resolution using PALM and EGFP- histones. PLoS One.

[31] Belmont A.S., Sedat J.W., Agard D.A. (1987). A three-dimensional approach to mitotic chromosome structure: Evidence for a complex heirarchial organization. J. Cell Biol..

[32] Belmont A.S., Braunfeld M.B., Sedat J.W., Agard D.A. (1989). Large-scale chromatin structural domains within mitotic and interphase chromosomes in vivo and in vitro. Chromosoma.

[33] Belmont A.S., Bruce K. (1994). Visualization of G1 chromosomes: a folded, twisted, supercoiled chromonema model of interphase chromatid structure. J. Cell Biol..

[34] Belmont A.S., Dietzel S., Nye A.C., Strukov Y.G., Tumbar T. (1999). Large-scale chromatin structure and function. Curr. Opin. Cell Biol..

[35] Nozaki T., Imai R., Tanbo M., Nagashima R., Tamura S., Tani T., Joti Y., Tomita M., Hibino K., Kanemaki M.T., Wendt K.S., Okada Y., Nagai T., Maeshima K. (2017). Dynamic organization of chromatin domains revealed by super-resolution live-cell imaging. Mol. Cell.

[36] Xu J., Ma H., Jin J., Uttam S., Fu R., Huang Y., Liu Y. (2018). Super-resolution imaging of higher-order chromatin structures at different epigenomic states in single mammalian cells. Cell Rep..

[37] Eltsov M., MacLellan K.M., Maeshima K., Frangakis A.S., Dubochet J. (2008). Analysis of cryo-electron microscopy images does not support the existence of 30-nm chromatin fibers in mitotic chromosomes in situ. Proc. Natl. Acad. Sci. U. S. A..

[38] Brangwynne C.P., Eckmann C.R., Courson D.S., Rybarska A., Hoege C., Gharakhani J., Julicher F., Hyman A.A. (2009). Germline P granules are liquid droplets that localize by controlled dissolution/condensation. Science.

[39] Balbiani E.G. (1881). Sur la structure du noyau des cellules salivaires chez les larves de chironomus. Zool. Anz..

[40] Wilson E.B. (1911). The Cell in Development and Inheritance.

[41] Bak A.L., Zeuthen J., Crick F.H.C. (1977). Higher-order structure of human mitotic chromosomes. Proc. Nat. Acad. Sci..

[42] Darlington C.D. (1935). The internal mechanics of the chromosomes. I - The nuclear cycle in Fritillaria. Proc. Roy. Soc. Lond. Ser. B.

[43] Kuwada Y. (1939). Chromosome structure. A critical review. Cytologia.

[44] Paulson J.R., Laemmli U.K. (1977). The structure of histone-depleted chromosomes. Cell.

[45] DuPraw E.J. (1966). Evidence for a “folded-fibre” organization in human chromosomes. Nature.

[46] Houchmandzadeh B., Marko J.F., Chatenay D., Libchaber A. (1997). Elasticity and structure of eukaryote chromosomes studied by micromanipulation and micropipette aspiration. J. Cell Biol..

[47] Poirier M.G., Eroglu S., Marko J.F. (2002). The bending rigidity of mitotic chromosomes. Mol. Biol. Cell.

[48] Poirier M.G., Marko J.F. (2003). Micromechanical studies of mitotic chromosomes. Curr. Top. Dev. Biol..

[49] Ohnuki Y. (1968). Structure of chromosomes. I. Morphological studies of the spiral structure of human somatic chromosomes. Chromosoma.

[50] Almagro S., Riveline D., Hirano T., Houchmandzadeh B., Dimitrov S. (2004). The mitotic chromosome is an assembly of rigid elastic axes organized by structural maintenance of chromosomes (SMC) proteins and surrounded by a soft chromatin envelope. J. Biol. Chem..

[51] Liang Z., Zickler D., Prentiss M., Chang F.S., Witz G., Maeshima K., Kleckner N. (2015). Chromosomes progress to metaphase in multiple discrete steps via global compaction/expansion cycles. Cell.

[52] Chu L., Liang Z., Mukhina M., Fisher J., Vincenten N., Zhang Z., Hutchinson J., Zickler D., Kleckner N. (2020). The 3D topography of mitotic chromosomes. Mol. Cell.

[53] Chu L., Liang Z., Mukhina M.V., Fisher J.K., Hutchinson J.W., Kleckner N.E. (2020). One-dimensional spatial patterning along mitotic chromosomes: a mechanical basis for macroscopic morphogenesis. Proc. Natl. Acad. Sci. USA.

[54] Earnshaw W.C. (1991). Large scale chromosome structure and organization. Curr. Opin. Struct. Biol..

[55] Swedlow J.R., Hirano T. (2003). The making of the mitotic chromosome: modern insights into classical questions. Mol. Cell.

[56] Gassmann R. (2004). Mitotic chromosome formation and the condensin paradox. Exp. Cell Res..

[57] Hudson D.F., Marshall K.M., Earnshaw W.C. (2009). Condensin: architect of mitotic chromosomes. Chromosome Res..

[58] Ohta S., Wood L., Bukowski-Wills J.C., Rappsilber J., Earnshaw W.C. (2010). Building mitotic chromosomes. Curr. Opin. Cell Biol..

[59] Vagnarelli P. (2012). Mitotic chromosome condensation in vertebrates. Exp. Cell Res..

[60] Dekker J. (2014). Two ways to fold the genome during the cell cycle: insights obtained with chromosome conformation capture. Epigenetics Chromatin.

[61] Hirano T. (2015). Chromosome dynamics during mitosis. Cold Spring Harb. Perspect. Biol..

[62] Dekker J., Mirny L. (2016). The 3D genome as moderator of chromosomal communication. Cell.

[63] Hirano T. (2016). Condensin-based chromosome organization from bacteria to vertebrates. Cell.

[64] Kalitsis P., Zhang T., Marshall K.M., Nielsen C.F., Hudson D.F. (2017). Condensin, master organizer of the genome. Chromosome Res..

[65] Takahashi M., Hirota T. (2019). Folding the genome into mitotic chromosomes. Curr. Opin. Cell Biol..

[66] Batty P., Gerlich D.W. (2019). Mitotic chromosome mechanics: how cells segregate their genome. Trends Cell Biol..

[67] MacGregor I.A., Adams I.R., Gilbert N. (2019). Large-scale chromatin organisation in interphase, mitosis and meiosis. Biochem J..

[68] Zhou C.Y., Heald R. (2020). Emergent properties of mitotic chromosomes. Curr. Opin. Cell Biol..

[69] Booth D.G., Takagi M., Sanchez-Pulido L., Petfalski E., Vargiu G., Samejima K., Imamoto N., Ponting C.P., Tollervey D., Earnshaw W.C., Vagnarelli P. (2014). Ki-67 is a PP1-interacting protein that organises the mitotic chromosome periphery. Elife.

[70] Cuylen S., Blaukopf C., Politi A.Z., Müller-Reichert T., Neumann B., Poser I., Ellenberg J., Hyman A.A., Gerlich D.W. (2016). Ki-67 acts as a biological surfactant to disperse mitotic chromosomes. Nature.

[71] Booth D.G., Earnshaw W.C. (2017). Ki-67 and the chromosome periphery compartment in mitosis. Trends Cell Biol..

[72] Takagi M., Natsume T., Kanemaki M.T., Imamoto N. (2016). Perichromosomal protein Ki67 supports mitotic chromosome architecture. Genes Cells.

[73] Takagi M., Ono T., Natsume T., Sakamoto C., Nakao M., Saitoh N., Kanemaki M.T., Hirano T., Imamoto N. (2018). Ki-67 and condensins support the integrity of mitotic chromosomes through distinct mechanisms. J. Cell Sci..

[74] Sobecki M., Mrouj K., Camasses A., Parisis N., Nicolas E., Llères D., Gerbe F., Prieto S., Krasinska L., David A., Eguren M., Birling M.C., Urbach S., Hem S., Déjardin J., Malumbres M., Jay P., Dulic V., Lafontaine D.L., Feil R., Fisher D. (2016). The cell proliferation antigen Ki-67 organises heterochromatin. Elife.

[75] Olins D.E., Olins A.L. (2018). Epichromatin and chromomeres: a ‘fuzzy’ perspective. Open Biol..

[76] Baranetzky J. (1880). Die Kerntheilung in den Pollen-mutterzellen einiger Tradescantien. Bot. Ztg..

[77] Vejdovský F. (1912).

[78] Kuwada Y. (1927). On the spiral structure of chromosomes. Bot. Mag..

[79] Telezynski H. (1930). Le Cycle du chromosome somatique, II. Acta Soc. Bot. Pol..

[80] Manton I. (1950). The spiral structure of chromosomes. Biol. Rev. Camb. Philos. Soc..

[81] Taylor J.H. (1958). The duplication of chromosomes. Sci. Am..

[82] Darlington C.D. (1955). The chromosome as a physico-chemical entity. Nature.

[83] Nokkala S., Nokkala C. (1985). Spiral structures of meiotic chromosomes in plants. Hereditas.

[84] Ruzicka F. (1974). Organization of human mitotic chromosomes. Humangenetik.

[85] Rattner J.B., Lin C.C. (1985). Radial loops and helical coils coexist in metaphase chromosomes. Cell.

[86] Stubblefield E., Wray W. (1971). Architecture of the Chinese hamster metaphase chromosome. Chromosoma.

[87] Earnshaw W.C., Laemmli U.K. (1983). Architecture of metaphase chromosomes and chromosome scaffolds. J. Cell Biol..

[88] Paulson J.R. (1989). Scaffold morphology in histone-depleted HeLa metaphase chromosomes. Chromosoma.

[89] Gibcus J.H., Samejima K., Goloborodko A., Samejima I., Naumova N., Nuebler J., Kanemaki M.T., Xie L., Paulson J.R., Earnshaw W.C., Mirny L.A., Dekker J. (2018). A pathway for mitotic chromosome formation. Science.

[90] Naumova N., Imakaev M., Fudenberg G., Zhan Y., Lajoie B.R., Mirny L.A., Dekker J. (2013). Organization of the mitotic chromosome. Science.

[91] Boy de la Tour E., Laemmli U.K. (1988). The metaphase scaffold is helically folded: sister chromatids have predominantly opposite helical handedness. Cell.

[92] DuPraw E.J. (1970).

[93] Caspersson T., Zech L., Johansson C. (1970). Analysis of human metaphase chromosome set by aid of DNA-binding fluorescent agents. Exp. Cell Res..

[94] Schnedl W. (1971). Banding pattern of human chromosomes. Nat. New Biol..

[95] Sumner A.T., Evans H.J., Buckland R.A. (1971). New technique for distinguishing between human chromosomes. Nat. New Biol..

[96] Patil S.R., Merrick S., Lubs H.A. (1971). Identification of each human chromosome with a modified Giemsa stain. Science.

[97] Drets M.E., Shaw M.W. (1971). Specific banding patterns of human chromosomes. Proc. Natl. Acad. Sci. USA.

[98] Huberman J.A. (1973). Structure of chromosome fibers and chromosomes. Annu. Rev. Biochem..

[99] Bostock C.J., Sumner A.T. (1978).

[100] Cuny G., Soriano P., Macaya G., Bernardi G. (1981). The major components of the mouse and human genomes. 1. Preparation, basic properties and compositional heterogeneity. Eur. J. Biochem..

[101] Costantini M., Musto H. (2017). The isochores as a fundamental level of genome structure and organization: a general overview. J. Mol. Evol..

[102] Bernardi G. (2019). The genomic code: a pervasive encoding/molding of chromatin structures and a solution of the “non‐coding DNA” mystery. Bioessays.

[103] Dutrillaux B., Couturier J., Viegas-Pequignot E., Bennett D.M., Bobrow M., Hewitt G. (1981). Chromosomes Today.

[104] Ferguson-Smith M.A., Trifonov V. (2007). Mammalian karyotype evolution. Nat. Rev. Genet..

[105] Adolph K.W., Cheng S.M., Laemmli U.K. (1977). Role of nonhistone proteins in metaphase chromosome structure. Cell.

[106] Earnshaw W.C., Laemmli U.K. (1984). Silver staining the chromosome scaffold. Chromosoma.

[107] Adolph K.W., Cheng S.M., Paulson J.R., Laemmli U.K. (1977). Isolation of a protein scaffold from mitotic HeLa cell chromosomes. Proc. Natl. Acad. Sci..

[108] Laemmli U.K., Cheng S.M., Adolph K.W., Paulson J.R., Brown J.A., Baumbach W.R. (1978). Metaphase chromosome structure: the role of nonhistone proteins. Cold Spring Harb. Symp. Quant. Biol..

[109] Adolph K.W. (1980). Organization of chromosomes in mitotic HeLa cells. Exp. Cell Res..

[110] Adolph K.W. (1981). A serial sectioning study of the structure of human mitotic chromosomes. Eur. J. Cell Biol..

[111] Jackson D.A., Dickinson P., Cook P.R. (1990). The size of chromatin loops in HeLa cells. EMBO J..

[112] Foe V.E., Akai H., King R. (1982). Insect Ultrastructure.

[113] Rattner J.B., Goldsmith M., Hamkalo B.A. (1980). Chromatin organization during meiotic prophase of Bombyx mori. Chromosoma.

[114] Rattner J.B., Goldsmith M.R., Hamkalo B.A. (1981). Chromosome organization during male meiosis in Bombyx mori. Chromosoma.

[115] Flemming W. (1882).

[116] Rückert J. (1892). Zur Entwicklungsgeschichte des Ovarialeies bei Selachiern. Anat. Anz..

[117] Ris H. (1945). The structure of meiotic chromosomes in the grasshopper and its bearing on the nature of “chromomeres” and “lampbrush chromosomes”. Biol. Bull..

[118] Lafontaine J.G., Ris H. (1958). An electron microscope study of lampbrush chromosomes. J. Biophys. Biochem. Cytol..

[119] Callan H.G., Lloyd L. (1960). Lampbrush chromosomes of crested newts Triturus cristatus (Laurenti). Philos. Trans. R. Soc. B.

[120] Lamb M.M., Daneholt B. (1979). Characterization of active transcription units in Balbiani rings of Chironomus tentans. Cell.

[121] Berezney R., Coffey D.S. (1974). Identification of a nuclear protein matrix. Biochem. Biophys. Res. Commun..

[122] Belgrader P., Siegel A.J., Berezney R. (1991). A comprehensive study on the isolation and characterization of the HeLa S3 nuclear matrix. J. Cell Sci..

[123] Okada T.A., Comings D.E. (1980). A search for protein cores in chromosomes: is the scaffold an artifact?. Am. J. Hum. Genet..

[124] Poirier M.G., Marko J.F. (2002). Mitotic chromosomes are chromatin networks without a mechanically contiguous protein scaffold. Proc. Natl. Acad. Sci. U. S. A..

[125] Earnshaw W.C., Heck M.M.S. (1985). Localization of topoisomerase II in mitotic chromosomes. J. Cell Biol..

[126] Mullinger A.M., Johnson R.T. (1979). The organization of supercoiled DNA from human chromosomes. J. Cell Sci..

[127] Earnshaw W.C., Halligan B., Cooke C.A., Heck M.M., Liu L.F. (1985). Topoisomerase II is a structural component of mitotic chromosome scaffolds. J. Cell Biol..

[128] Gasser S.M., Laroche T., Falquet J., Boy de la Tour E., Laemmli U.K. (1986). Metaphase chromosome structure. Involvement of topoisomerase II. J. Mol. Biol..

[129] Saitoh N., Goldberg I.G., Wood E.R., Earnshaw W.C. (1994). ScII: an abundant chromosome scaffold protein is a member of a family of putative ATPases with an unusual predicted tertiary structure. J. Cell Biol..

[130] Hirano T., Mitchison T.J. (1994). A heterodimeric coiled-coil protein required for mitotic chromosome condensation in vitro. Cell.

[131] Hirano T., Kobayashi R., Hirano M. (1997). Condensins, chromosome condensation protein complexes containing XCAP-C, XCAP-E and a Xenopus homolog of the Drosophila Barren protein. Cell.

[132] Hudson D.F., Vagnarelli P., Gassmann R., Earnshaw W.C. (2003). Condensin is required for nonhistone protein assembly and structural integrity of vertebrate mitotic chromosomes. Dev. Cell.

[133] Samejima K., Samejima I., Vagnarelli P., Ogawa H., Vargiu G., Kelly D.A., de Lima Alves F., Kerr A., Green L.C., Hudson D.F., Ohta S., Cooke C.A., Farr C.J., Rappsilber J., Earnshaw W.C. (2012). Mitotic chromosomes are compacted laterally by KIF4 and condensin and axially by topoisomerase IIalpha. J. Cell Biol..

[134] Samejima K., Booth D.G., Ogawa H., Paulson J.R., Xie L., Watson C.A., Platani M., Kanemaki M.T., Earnshaw W.C. (2018). Functional analysis after rapid degradation of condensins and 3D-EM reveals chromatin volume is uncoupled from chromosome architecture in mitosis. J. Cell Sci..

[135] Nielsen C.F., Zhang T., Barisic M., Kalitsis P., Hudson D.F. (2020). Topoisomerase IIalpha is essential for maintenance of mitotic chromosome structure. Proc. Natl. Acad. Sci. USA.

[136] Koshland D., Strunnikov A. (1996). Mitotic chromosome condensation. Annu. Rev. Cell Dev. Biol..

[137] Hagstrom K.A., Meyer B.J. (2003). Condensin and cohesin: more than chromosome compactor and glue. Nat. Rev. Genet..

[138] Belmont A.S. (2006). Mitotic chromosome structure and condensation. Curr. Opin. Cell Biol..

[139] Maeshima K., Eltsov M. (2008). Packaging the genome: the structure of mitotic chromosomes. J. Biochem..

[140] Marko J.F. (2008). Micromechanical studies of mitotic chromosomes. Chromosome Res..

[141] Yanagida M. (2009). Clearing the way for mitosis: is cohesin a target?. Nat. Rev. Mol. Cell Biol..

[142] Baxter J., Aragon L. (2011). A model for chromosome condensation based on the interplay between condensin and topoisomerase II. Trends Genet..

[143] Moser S.C., Swedlow J.R. (2011). How to be a mitotic chromosome. Chromosome Res..

[144] Ohta S., Wood L., Bukowski-Wills J.C., Rappsilber J., Earnshaw W.C. (2011). Building mitotic chromosomes. Curr. Opin. Cell Biol..

[145] Peters J.M., Nishiyama T. (2012). Sister chromatid cohesion. Cold Spring Harb. Perspect. Biol..

[146] Aragon L., Martinez-Perez E., Merkenschlager M. (2013). Condensin, cohesin and the control of chromatin states. Curr. Opin. Genet. Dev..

[147] Jeppsson K., Kanno T., Shirahige K., Sjögren C. (2014). The maintenance of chromosome structure: positioning and functioning of SMC complexes. Nat. Rev. Mol. Cell. Biol..

[148] Kschonsak M., Haering C.H. (2015). Shaping mitotic chromosomes: from classical concepts to molecular mechanisms. Bioessays.

[149] Piskadlo E., Oliveira R.A. (2017). A topology-centric view on mitotic chromosome architecture. Int. J. Mol. Sci..

[150] Kinoshita K., Hirano T. (2017). Dynamic organization of mitotic chromosomes. Curr. Opin. Cell Biol..

[151] van Wely K.H.M., Mora Gallardo C., Vann K.R., Kutateladze T.G. (2017). Epigenetic countermarks in mitotic chromosome condensation. Nucleus.

[152] Almeida A.C., Maiato H. (2018). Chromokinesins. Curr. Biol..

[153] Kakui Y., Uhlmann F. (2018). SMC complexes orchestrate the mitotic chromatin interaction landscape. Curr. Genet..

[154] Yusuf M., Kaneyoshi K., Fukui K., Robinson I. (2019). Use of 3D imaging for providing insights into high-order structure of mitotic chromosomes. Chromosoma.

[155] Mirny L.A., Imakaev M., Abdennur N. (2019). Two major mechanisms of chromosome organization. Curr. Opin. Cell Biol..

[156] Sedeno Cacciatore, A. and B. D. Rowland (2019). Loop formation by SMC complexes: turning heads, bending elbows, and fixed anchors. Curr. Opin. Genet. Dev..

[157] Yatskevich S., Rhodes J., Nasmyth K. (2019). Organization of chromosomal DNA by SMC complexes. Annu. Rev. Genet..

[158] Beseda T., Cápal P., Kubalová I., Schubert V., Doležel J., Šimková H. (2020). Mitotic chromosome organization: general rules meet species-specific variability. Comput. Struct. Biotechnol. J..

[159] Makela J., Sherratt D. (2020). SMC complexes organize the bacterial chromosome by lengthwise compaction. Curr. Genet..

[160] Datta S., Lecomte L., Haering C.H. (2020). Structural insights into DNA loop extrusion by SMC protein complexes. Curr. Opin. Struct. Biol..

[161] Hassler M., Shaltiel I.A., Haering C.H. (2018). Towards a unified model of SMC complex function. Curr. Biol..

[162] Cole A. (1967). Chromosome structure. Theor. Biophys..

[163] Laemmli U.K., Lewis C.D., Earnshaw W.C. (1983). Cancer: From Molecules to Man.

[164] Shintomi K., Inoue F., Watanabe H., Ohsumi K., Ohsugi M., Hirano T. (2017). Mitotic chromosome assembly despite nucleosome depletion in Xenopus egg extracts. Science.

[165] Booth D.G., Beckett A.J., Molina O., Samejima I., Masumoto H., Kouprina N., Larionov V., Prior I.A., Earnshaw W.C. (2016). 3D-CLEM reveals that a major portion of mitotic chromosomes is not chromatin. Mol. Cell.

[166] Ngo J.T., Adams S.R., Deerinck T.J., Boassa D., Rodriguez-Rivera F., Palida S.F., Bertozzi C.R., Ellisman M.H., Tsien R.Y. (2016). Click-EM for imaging metabolically tagged nonprotein biomolecules. Nat. Chem. Biol..

[167] Poonperm R., Takata H., Hamano T., Matsuda A., Uchiyama S., Hiraoka Y., Fukui K. (2015). Chromosome scaffold is a double-stranded assembly of scaffold proteins. Sci. Rep..

[168] Rust M.J., Bates M., Zhuang X. (2006). Sub-diffraction-limit imaging by stochastic optical reconstruction microscopy (STORM). Nat. Methods.

[169] Chen F., Tillberg P.W., Boyden E.S. (2015). Optical imaging expansion microscopy. Science.

[170] Xu H., Tong Z., Ye Q., Sun T., Hong Z., Zhang L., Bortnick A., Cho S., Beuzer P., Axelrod J., Hu Q., Wang M., Evans S.M., Murre C., Lu L.F., Sun S., Corbett K.D., Cang H. (2019). Molecular organization of mammalian meiotic chromosome axis revealed by expansion STORM microscopy. Proc. Natl. Acad. Sci. USA.

[171] Ohta S., Bukowski-Wills J.C., Sanchez-Pulido L., Alves F.L., Wood L., Chen Z.A., Platani M., Fischer L., Hudson D.F., Ponting C.P., Fukagawa T., Earnshaw W.C., Rappsilber J. (2010). The protein composition of mitotic chromosomes determined using multiclassifier combinatorial proteomics. Cell.

[172] Fukagawa T., Earnshaw W.C. (2014). The centromere: chromatin foundation for the kinetochore machinery. Dev. Cell.

[173] Pesenti M.E., Weir J.R., Musacchio A. (2016). Progress in the structural and functional characterization of kinetochores. Curr. Opin. Struct. Biol..

[174] McKinley K.L., Cheeseman I.M. (2016). The molecular basis for centromere identity and function. Nat. Rev. Mol. Cell Biol..

[175] Samejima I., Platani M., Earnshaw W.C. (2017). Use of mass spectrometry to study the centromere and kinetochore. Prog. Mol. Subcell. Biol..

[176] Hara M., Fukagawa T. (2018). Kinetochore assembly and disassembly during mitotic entry and exit. Curr. Opin. Cell Biol..

[177] Hamilton G., Dimitrova Y., Davis T.N. (2019). Seeing is believing: our evolving view of kinetochore structure, composition, and assembly. Curr. Opin. Cell Biol..

[178] Ohzeki J., Larionov V., Earnshaw W.C., Masumoto H. (2019). De novo formation and epigenetic maintenance of centromere chromatin. Curr. Opin. Cell Biol..

[179] Hara M., Fukagawa T. (2020). Dynamics of kinetochore structure and its regulations during mitotic progression. Cell Mol. Life Sci..

[180] Morrison C. (2002). Proteomic analysis of human metaphase chromosomes reveals topoisomerase II alpha as an Aurora B substrate. Nucleic Acids Res..

[181] Gassmann R., Henzing A.J., Earnshaw W.C. (2005). Novel components of human mitotic chromosomes identified by proteomic analysis of the chromosome scaffold fraction. Chromosoma.

[182] Uchiyama S., Kobayashi S., Takata H., Ishihara T., Hori N., Higashi T., Hayashihara K., Sone T., Higo D., Nirasawa T., Takao T., Matsunaga S., Fukui K. (2005). Proteome analysis of human metaphase chromosomes. J. Biol. Chem..

[183] Fukui K., Uchiyama S. (2007). Chromosome protein framework from proteome analysis of isolated human metaphase chromosomes. Chem. Rec..

[184] Takata H., Uchiyama S., Nakamura N., Nakashima S., Kobayashi S., Sone T., Kimura S., Lahmers S., Granzier H., Labeit S., Matsunaga S., Fukui K. (2007). A comparative proteome analysis of human metaphase chromosomes isolated from two different cell lines reveals a set of conserved chromosome-associated proteins. Genes Cells.

[185] Kustatscher G., Wills K.L.H., Furlan C., Rappsilber J. (2014). Chromatin enrichment for proteomics. Nat. Protoc..

[186] Ohta S., Montaño-Gutierrez L.F., de Lima Alves F., Ogawa H., Toramoto I., Sato N., Morrison C.G., Takeda S., Hudson D.F., Rappsilber J., Earnshaw W.C. (2016). Proteomics analysis with a nano random forest approach reveals novel functional interactions regulated by SMC complexes on mitotic chromosomes. Mol. Cell Proteom..

[187] Ohta S., Kimura M., Takagi S., Toramoto I., Ishihama Y. (2016). Identification of mitosis-specific phosphorylation in mitotic chromosome-associated proteins. J. Proteome Res..

[188] Ohta S., Taniguchi T., Sato N., Hamada M., Taniguchi H., Rappsilber J. (2019). Quantitative proteomics of the mitotic chromosome scaffold reveals the association of BAZ1B with chromosomal axes. Mol. Cell Proteom..

[189] Ohta S., Bukowski-Wills J.C., Wood L., de Lima Alves F., Chen Z., Rappsilber J., Earnshaw W.C. (2010). Proteomics of isolated mitotic chromosomes identifies the kinetochore protein Ska3/Rama1. Cold Spring Harb. Symp. Quant. Biol..

[190] Stenström L., Mahdessian D., Gnann C., Cesnik A.J., Ouyang W., Leonetti M.D., Uhlén M., Cuylen‐Haering S., Thul P.J., Lundberg E. (2020). Mapping the nucleolar proteome reveals a spatiotemporal organization related to intrinsic protein disorder. Mol. Syst. Biol..

[191] Lewis C.D., Laemmli U.K. (1982). Higher order metaphase chromosome structure: evidence for metalloprotein interactions. Cell.

[192] Heck M.M.S., Earnshaw W.C. (1986). Topoisomerase II: a specific marker for proliferating cells. J. Cell Biol..

[193] Uemura T., Yanagida M. (1984). Isolation of type I and II DNA topoisomerase mutants from fission yeast: single and double mutants show different phenotypes in cell growth and chromatin organization. EMBO J..

[194] Gorbsky G.J. (1994). Cell cycle progression and chromosome segregation in mammalian cells cultured in the presence of the topoisomerase II inhibitors ICRF-187 [(+)-1,2-bis(3,5-dioxopiperazinyl-1-yl)propane; ADR-529] and ICRF-159 (Razoxane). Cancer Res..

[195] Chang C.J. (2003). RNAi analysis reveals an unexpected role for topoisomerase II in chromosome arm congression to a metaphase plate. J. Cell Sci..

[196] Saitoh Y., Laemmli U.K. (1994). Metaphase chromosome structure: bands arise from a differential folding path of the high AT-rich scaffold. Cell.

[197] Tavormina P.A., Côme M.G., Hudson J.R., Mo Y.Y., Beck W.T., Gorbsky G.J. (2002). Rapid exchange of mammalian topoisomerase II alpha at kinetochores and chromosome arms in mitosis. J. Cell Biol..

[198] Swedlow J.R., Sedat J.W., Agard D.A. (1993). Multiple chromosomal populations of topoisomerase II detected in vivo by time-lapse, three dimensional wide-field microscopy. Cell.

[199] Christensen M.O., Larsen M.K., Barthelmes H.U., Hock R., Andersen C.L., Kjeldsen E., Knudsen B.R., Westergaard O., Boege F., Mielke C. (2002). Dynamics of human DNA topoisomerases IIalpha and IIbeta in living cells. J. Cell Biol..

[200] Tavormina P.A., Côme M.G., Hudson J.R., Mo Y.Y., Beck W.T., Gorbsky G.J. (2002). Rapid exchange of mammalian topoisomerase II alpha at kinetochores and chromosome arms in mitosis. J. Cell Biol..

[201] Adachi Y., Luke M., Laemmli U.K. (1991). Chromosome assembly in vitro: topoisomerase II is required for condensation. Cell.

[202] Hirano T., Mitchison T.J. (1993). Topoisomerase II does not play a scaffolding role in the organization of mitotic chromosomes assembled in Xenopus egg extracts. J. Cell Biol..

[203] Cuvier O., Hirano T. (2003). A role of topoisomerase II in linking DNA replication to chromosome condensation. J. Cell Biol..

[204] Ishida R. (1991). Inhibition of intracellular topoisomerase II by antitumor bis(2,6- dioxopiperazine) derivatives: mode of cell growth inhibition distinct from that of cleavable complex-forming type inhibitors. Cancer Res..

[205] Anderson H., Roberge M. (1996). Topoisomerase II inhibitors affect entry into mitosis and chromosome condensation in BHK cells. Cell Growth Differ..

[206] Farr C.J., Antoniou-Kourounioti M., Mimmack M.L., Volkov A., Porter A.C.G. (2014). The alpha isoform of topoisomerase II is required for hypercompaction of mitotic chromosomes in human cells. Nucleic Acids Res..

[207] Ono T., Losada A., Hirano M., Myers M.P., Neuwald A.F., Hirano T. (2003). Differential contributions of condensin I and condensin II to mitotic chromosome architecture in vertebrate cells. Cell.

[208] Green L.C., Kalitsis P., Chang T.M., Cipetic M., Kim J.H., Marshall O., Turnbull L., Whitchurch C.B., Vagnarelli P., Samejima K., Earnshaw W.C., Choo K.H.A., Hudson D.F. (2012). Contrasting roles of condensin I and condensin II in mitotic chromosome formation. J. Cell Sci..

[209] Ono T., Sakamoto C., Nakao M., Saitoh N., Hirano T. (2017). Condensin II plays an essential role in reversible assembly of mitotic chromosomes in situ. Mol. Biol. Cell.

[210] Piskadlo E., Tavares A., Oliveira R.A. (2017). Metaphase chromosome structureis dynamically maintained by condensin I-directed DNA (de)catenation. Elife.

[211] Guacci V., Yamamoto A., Strunnikov A., Kingsbury J., Hogan E., Meluh P., Koshland D. (1993). Structure and function of chromosomes in mitosis of budding yeast. Cold Spring Harb. Symp. Quant. Biol..

[212] Strunnikov A.V., Larionov V.L., Koshland D. (1993). SMC1: an essential yeast gene encoding a putative head-rod-tail protein is required for nuclear division and defines a new ubiquitous protein family. J. Cell Biol..

[213] Saka Y., Sutani T., Yamashita Y., Saitoh S., Takeuchi M., Nakaseko Y., Yanagida M. (1994). Fission yeast cut3 and cut14, members of a ubiquitous protein family, are required for chromosome condensation and segregation in mitosis. EMBO J..

[214] Chuang P.-T., Albertson D.G., Meyer B.J. (1994). DPY-27: a chromosome condensation protein homolog that regulates C. elegans dosage compensation through association with the X chromosome. Cell.

[215] Schmiesing J.A., Gregson H.C., Zhou S., Yokomori K. (2000). A human condensin complex containing hCAP-C-hCAP-E and CNAP1, a homolog of Xenopus XCAP-D2, colocalizes with phosphorylated histone H3 during the early stage of mitotic chromosome condensation. Mol. Cell Biol..

[216] Hirano T. (2012). Condensins: universal organizers of chromosomes with diverse functions. Genes Dev..

[217] Aragon L. (2018). The Smc5/6 complex: new and old functions of the enigmatic long-distance relative. Annu. Rev. Genet..

[218] Larionov V.L., Karpova T.S., Kouprina N.Y., Jouravleva G.A. (1985). A mutant of Saccharomyces cerevisiae with impaired maintenance of centromeric plasmids. Curr. Genet..

[219] Gruber S. (2018). SMC complexes sweeping through the chromosome: going with the flow and against the tide. Curr. Opin. Microbiol..

[220] Makela J., Sherratt D.J. (2020). Organization of the Escherichia coli chromosome by a MukBEF axial core. Mol. Cell.

[221] Sakai A. (2003). Condensin but not cohesin SMC heterodimer induces DNA reannealing through protein-protein assembly. EMBO J..

[222] Kimura K., Hirano T. (1997). ATP-dependent positive supercoiling of DNA by 13S condensin: a biochemical implication for chromosome condensation. Cell.

[223] Bazett-Jones D.P., Kimura K., Hirano T. (2002). Efficient supercoiling of DNA by a single condensin complex as revealed by electron spectroscopic imaging. Mol. Cell.

[224] Ono T., Fang Y., Spector D.L., Hirano T. (2004). Spatial and temporal regulation of Condensins I and II in mitotic chromosome assembly in human cells. Mol. Biol. Cell.

[225] Hirota T. (2004). Distinct functions of condensin I and II in mitotic chromosome assembly. J. Cell Sci..

[226] Hagstrom K.A. (2002). C. elegans condensin promotes mitotic chromosome architecture, centromere organization, and sister chromatid segregation during mitosis and meiosis. Genes Dev..

[227] Csankovszki G., Collette K., Spahl K., Carey J., Snyder M., Petty E., Patel U., Tabuchi T., Liu H., McLeod I., Thompson J., Sarkesik A., Yates J., Meyer B.J., Hagstrom K. (2009). Three distinct condensin complexes control C. elegans chromosome dynamics. Curr. Biol..

[228] Crane E., Bian Q., McCord R.P., Lajoie B.R., Wheeler B.S., Ralston E.J., Uzawa S., Dekker J., Meyer B.J. (2015). Condensin-driven remodelling of X chromosome topology during dosage compensation. Nature.

[229] Coelho P.A., Queiroz-Machado J., Sunkel C.E. (2003). Condensin-dependent localisation of topoisomerase II to an axial chromosomal structure is required for sister chromatid resolution during mitosis. J. Cell Sci..

[230] Savvidou E. (2005). Drosophila CAP-D2 is required for condensin complex stability and resolution of sister chromatids. J. Cell Sci..

[231] D’Amours D., Stegmeier F., Amon A. (2004). Cdc14 and condensin control the dissolution of cohesin-independent chromosome linkages at repeated DNA. Cell.

[232] Bhat M.A., Philp A.V., Glover D.M., Bellen H.J. (1996). Chromatid segregation at anaphase requires the barren product, a novel chromosome-associated protein that interacts with Topoisomerase II. Cell.

[233] Steffensen S., Coelho P.A., Cobbe N., Vass S., Costa M., Hassan B., Prokopenko S.N., Bellen H., Heck M.M.S., Sunkel C.E. (2001). A role for Drosophila SMC4 in the resolution of sister chromatids in mitosis. Curr. Biol..

[234] Saitoh N., Goldberg I.G., Earnshaw W.C. (1995). The SMC proteins and the coming of age of the chromosome scaffold hypothesis. BioEssays.

[235] Melby T.E., Ciampaglio C.N., Briscoe G., Erickson H.P. (1998). The symmetrical structure of structural maintenance of chromosomes (SMC) and MukB proteins: long, antiparallel coiled coils, folded at a flexible hinge. J. Cell Biol..

[236] Barysz H., Kim J.H., Chen Z.A., Hudson D.F., Rappsilber J., Gerloff D.L., Earnshaw W.C. (2015). Three-dimensional topology of the SMC2/SMC4 subcomplex from chicken condensin I revealed by cross-linking and molecular modelling. Open Biol..

[237] Diebold-Durand M.L., Lee H., Ruiz Avila L.B., Noh H., Shin H.C., Im H., Bock F.P., Bürmann F., Durand A., Basfeld A., Ham S., Basquin J., Oh B.H., Gruber S. (2017). Structure of full-length SMC and rearrangements required for chromosome organization. Mol. Cell.

[238] Shi Z., Gao H., Bai X., Yu H. (2020). Cryo-EM structure of the human cohesin-NIPBL-DNA complex. Science.

[239] Lee B.G., Merkel F., Allegretti M., Hassler M., Cawood C., Lecomte L., O’Reilly F.J., Sinn L.R., Gutierrez-Escribano P., Kschonsak M., Bravo S., Nakane T., Rappsilber J., Aragon L., Beck M., Löwe J., Haering C.H. (2020). Cryo-EM structures of holo condensin reveal a subunit flip-flop mechanism. Nat. Struct. Mol. Biol..

[240] Hopfner K.P., Tainer J.A. (2003). Rad50/SMC proteins and ABC transporters: unifying concepts from high-resolution structures. Curr. Opin. Struct. Biol..

[241] Hopfner K.P. (2016). Invited review: Architectures and mechanisms of ATP binding cassette proteins. Biopolymers.

[242] Cobbe N., Heck M.M. (2006). The evolution of ATPase activity in SMC proteins. Proteins.

[243] Elbatsh A.M.O., Kim E., Eeftens J.M., Raaijmakers J.A., van der Weide R.H., García-Nieto A., Bravo S., Ganji M., uit de Bos J., Teunissen H., Medema R.H., de Wit E., Haering C.H., Dekker C., Rowland B.D. (2019). Distinct roles for Condensin’s two ATPase sites in chromosome condensation. Mol. Cell.

[244] Cobbe N., Heck M.M. (2000). Review: SMCs in the world of chromosome biology- from prokaryotes to higher eukaryotes. J. Struct. Biol..

[245] Nasmyth K., Haering C.H. (2005). The structure and function of SMC and kleisin complexes. Annu. Rev. Biochem..

[246] Hirano T. (2006). At the heart of the chromosome: SMC proteins in action. Nat. Rev. Mol. Cell Biol..

[247] Onn I., Heidinger-Pauli J.M., Guacci V., Ünal E., Koshland D.E. (2008). Sister chromatid cohesion: a simple concept with a complex reality. Annu. Rev. Cell Dev. Biol..

[248] Uhlmann F. (2016). SMC complexes: from DNA to chromosomes. Nat. Rev. Mol. Cell Biol..

[249] Takahashi M., Wakai T., Hirota T. (2016). Condensin I-mediated mitotic chromosome assembly requires association with chromokinesin KIF4A. Genes Dev..

[250] Takemoto A., Maeshima K., Ikehara T., Yamaguchi K., Murayama A., Imamura S., Imamoto N., Yokoyama S., Hirano T., Watanabe Y., Hanaoka F., Yanagisawa J., Kimura K. (2009). The chromosomal association of condensin II is regulated by a noncatalytic function of PP2A. Nat. Struct. Mol. Biol..

[251] Wang X., Garvanska D.H., Nasa I., Ueki Y., Zhang G., Kettenbach A.N., Peti W., Nilsson J., Page R. (2020). A dynamic charge-charge interaction modulates PP2A:B56 substrate recruitment. Elife.

[252] Watrin E., Legagneux V. (2005). Contribution of hCAP-D2, a non-SMC subunit of condensin I, to chromosome and chromosomal protein dynamics during mitosis. Mol. Cell Biol..

[253] Baxter J., Sen N., Martinez V.L., De Carandini M.E.M., Schvartzman J.B., Diffley J.F.X., Aragon L. (2011). Positive supercoiling of mitotic DNA drives decatenation by topoisomerase II in eukaryotes. Science.

[254] Charbin A., Bouchoux C., Uhlmann F. (2014). Condensin aids sister chromatid decatenation by topoisomerase II. Nucleic Acids Res..

[255] Shintomi K., Takahashi T.S., Hirano T. (2015). Reconstitution of mitotic chromatids with a minimum set of purified factors. Nat. Cell Biol..

[256] Leonard J., Sen N., Torres R., Sutani T., Jarmuz A., Shirahige K., Aragón L. (2015). Condensin relocalization from centromeres to chromosome arms promotes Top2 recruitment during anaphase. Cell Rep..

[257] Sen N., Leonard J., Torres R., Garcia-Luis J., Palou-Marin G., Aragón L. (2016). Physical proximity of sister chromatids promotes Top2-dependent intertwining. Mol. Cell.

[258] Daniloski Z., Bisht K.K., McStay B., Smith S. (2019). Resolution of human ribosomal DNA occurs in anaphase, dependent on tankyrase 1, condensin II, and topoisomerase IIalpha. Genes Dev..

[259] Orlandini E., Marenduzzo D., Michieletto D. (2019). Synergy of topoisomerase and structural-maintenance-of-chromosomes proteins creates a universal pathway to simplify genome topology. Proc. Natl. Acad. Sci. USA.

[260] Dyson S., Segura J., Martínez‐García B., Valdés A., Roca J. (2021). Condensin minimizes topoisomerase II-mediated entanglements of DNA in vivo. EMBO J..

[261] Somma M.P. (2003). Chromosome condensation defects in barren RNA-interfered Drosophila cells. Genetics.

[262] Gerlich D., Hirota T., Koch B., Peters J.M., Ellenberg J. (2006). Condensin I stabilizes chromosomes mechanically through a dynamic interaction in live cells. Curr. Biol..

[263] Houlard M., Godwin J., Metson J., Lee J., Hirano T., Nasmyth K. (2015). Condensin confers the longitudinal rigidity of chromosomes. Nat. Cell Biol..

[264] Guacci V., Koshland D., Strunnikov A. (1997). A direct link between sister chromatid cohesion and chromosome condensation revealed through the analysis of MCD1 in S. cerevisiae. Cell.

[265] Michaelis C., Ciosk R., Nasmyth K. (1997). Cohesins: chromosomal proteins that prevent premature separation of sister chromatids. Cell.

[266] Uhlmann F., Nasmyth K. (1998). Cohesion between sister chromatids must be established during DNA replication. Curr. Biol..

[267] Haering C.H., Schoffnegger D., Nishino T., Helmhart W., Nasmyth K., Löwe J. (2004). Structure and stability of cohesin’s Smc1-kleisin interaction. Mol. Cell.

[268] Ström L., Lindroos H.B., Shirahige K., Sjögren C. (2004). Postreplicative recruitment of cohesin to double-strand breaks is required for DNA repair. Mol. Cell.

[269] Ünal E., Arbel-Eden A., Sattler U., Shroff R., Lichten M., Haber J.E., Koshland D. (2004). DNA damage response pathway uses histone modification to assemble a double-strand break-specific cohesin domain. Mol. Cell.

[270] Potts P.R., Porteus M.H., Yu H. (2006). Human SMC5/6 complex promotes sister chromatid homologous recombination by recruiting the SMC1/3 cohesin complex to double-strand breaks. EMBO J..

[271] Sumara I., Vorlaufer E., Gieffers C., Peters B.H., Peters J.M. (2000). Characterization of vertebrate cohesin complexes and their regulation in prophase. J. Cell Biol..

[272] Waizenegger I.C., Hauf S., Meinke A., Peters J.M. (2000). Two distinct pathways remove mammalian cohesin from chromosome arms in prophase and from centromeres in anaphase. Cell.

[273] Losada A., Hirano M., Hirano T. (2002). Cohesin release is required for sister chromatid resolution, but not for condensin-mediated compaction, at the onset of mitosis. Genes Dev..

[274] Hauf S., Roitinger E., Koch B., Dittrich C.M., Mechtler K., Peters J.M. (2005). Dissociation of cohesin from chromosome arms and loss of arm cohesion during early mitosis depends on phosphorylation of SA2. PLoS Biol..

[275] McGuinness B.E., Hirota T., Kudo N.R., Peters J.M., Nasmyth K. (2005). Shugoshin prevents dissociation of cohesin from centromeres during mitosis in vertebrate cells. PLoS Biol..

[276] Uhlmann F., Lottspeich F., Nasmyth K. (1999). Sister-chromatid separation at anaphase onset is promoted by cleavage of the cohesin subunit Scc1. Nature.

[277] Uhlmann F., Wernic D., Poupart M.A., Koonin E.V., Nasmyth K. (2000). Cleavage of cohesin by the CD clan protease separin triggers anaphase in yeast. Cell.

[278] Wirth K.G., Wutz G., Kudo N.R., Desdouets C., Zetterberg A., Taghybeeglu S., Seznec J., Ducos G.M., Ricci R., Firnberg N., Peters J.M., Nasmyth K. (2006). Separase: a universal trigger for sister chromatid disjunction but not chromosome cycle progression. J. Cell Biol..

[279] Nasmyth K. (2011). Cohesin: a catenase with separate entry and exit gates?. Nat. Cell Biol..

[280] Vagnarelli P., Morrison C., Dodson H., Sonoda E., Takeda S., Earnshaw W.C. (2004). Analysis of Scc1-deficient cells defines a key metaphase role of vertebrate cohesin in linking sister kinetochores. EMBO Rep..

[281] Schleiffer A., Kaitna S., Maurer-Stroh S., Glotzer M., Nasmyth K., Eisenhaber F. (2003). Kleisins: a superfamily of bacterial and eukaryotic SMC protein partners. Mol. Cell.

[282] Wells J.N., Gligoris T.G., Nasmyth K.A., Marsh J.A. (2017). Evolution of condensin and cohesin complexes driven by replacement of Kite by Hawk proteins. Curr. Biol..

[283] Ciosk R., Shirayama M., Shevchenko A., Tanaka T., Toth A., Shevchenko A., Nasmyth K. (2000). Cohesin’s binding to chromosomes depends on a separate complex consisting of Scc2 and Scc4 proteins. Mol. Cell.

[284] Murayama Y., Uhlmann F. (2014). Biochemical reconstitution of topological DNA binding by the cohesin ring. Nature.

[285] Rhodes J., Mazza D., Nasmyth K., Uphoff S. (2017). Scc2/Nipbl hops between chromosomal cohesin rings after loading. Elife.

[286] Hartman T., Stead K., Koshland D., Guacci V. (2000). Pds5p is an essential chromosomal protein required for both sister chromatid cohesion and condensation in Saccharomyces cerevisiae. J. Cell Biol..

[287] Panizza S., Tanaka T., Hochwagen A., Eisenhaber F., Nasmyth K. (2000). Pds5 cooperates with cohesin in maintaining sister chromatid cohesion. Curr. Biol..

[288] Vaur S., Feytout A., Vazquez S., Javerzat J.P. (2012). Pds5 promotes cohesin acetylation and stable cohesin-chromosome interaction. EMBO Rep..

[289] Rowland B.D., Roig M.B., Nishino T., Kurze A., Uluocak P., Mishra A., Beckouët F., Underwood P., Metson J., Imre R., Mechtler K., Katis V.L., Nasmyth K. (2009). Building sister chromatid cohesion: smc3 acetylation counteracts an antiestablishment activity. Mol. Cell.

[290] Minamino M., Ishibashi M., Nakato R., Akiyama K., Tanaka H., Kato Y., Negishi L., Hirota T., Sutani T., Bando M., Shirahige K. (2015). Esco1 acetylates cohesin via a mechanism different from that of Esco2. Curr. Biol..

[291] Rankin S., Ayad N.G., Kirschner M.W. (2005). Sororin, a substrate of the anaphase-promoting complex, is required for sister chromatid cohesion in vertebrates. Mol. Cell.

[292] Schmitz J., Watrin E., Lénárt P., Mechtler K., Peters J.M. (2007). Sororin is required for stable binding of cohesin to chromatin and for sister chromatid cohesion in interphase. Curr. Biol..

[293] Nishiyama T., Ladurner R., Schmitz J., Kreidl E., Schleiffer A., Bhaskara V., Bando M., Shirahige K., Hyman A.A., Mechtler K., Peters J.M. (2010). Sororin mediates sister chromatid cohesion by antagonizing Wapl. Cell.

[294] Gandhi R., Gillespie P.J., Hirano T. (2006). Human Wapl is a cohesin-binding protein that promotes sister-chromatid resolution in mitotic prophase. Curr. Biol..

[295] Sutani T., Kawaguchi T., Kanno R., Itoh T., Shirahige K. (2009). Budding yeast Wpl1(Rad61)-Pds5 complex counteracts sister chromatid cohesion-establishing reaction. Curr. Biol..

[296] Ouyang Z., Zheng G., Tomchick D.R., Luo X., Yu H. (2016). Structural basis and IP6 requirement for Pds5-dependent cohesin dynamics. Mol. Cell.

[297] Gillis L.A., McCallum J., Kaur M., DeScipio C., Yaeger D., Mariani A., Kline A.D., Li H., Devoto M., Jackson L.G., Krantz I.D. (2004). NIPBL mutational analysis in 120 individuals with Cornelia de Lange syndrome and evaluation of genotype-phenotype correlations. Am. J. Hum. Genet..

[298] Tonkin E.T., Wang T.J., Lisgo S., Bamshad M.J., Strachan T. (2004). NIPBL, encoding a homolog of fungal Scc2-type sister chromatid cohesion proteins and fly Nipped-B, is mutated in Cornelia de Lange syndrome. Nat. Genet..

[299] Strachan T. (2005). Cornelia de Lange Syndrome and the link between chromosomal function, DNA repair and developmental gene regulation. Curr. Opin. Genet. Dev..

[300] Liu J., Krantz I.D. (2008). Cohesin and human disease. Annu. Rev. Genom. Hum. Genet..

[301] McNairn A.J., Gerton J.L. (2008). Cohesinopathies: one ring, many obligations. Mutat. Res..

[302] Cheng H., Zhang N., Pati D. (2020). Cohesin subunit RAD21: from biology to disease. Gene.

[303] Freeman M.V.R., Williams D.W., Schimke R.N., Temtamy S.A., Vachier E., German J. (1974). The Roberts syndrome. Clin. Genet..

[304] German J. (1979). Roberts’ syndrome. I. Cytological evidence for a disturbance in chromatid pairing. Clin. Genet..

[305] Zakari M., Yuen K., Gerton J.L. (2015). Etiology and pathogenesis of the cohesinopathies. Wiley Interdiscip. Rev. Dev. Biol..

[306] Sarogni P., Pallotta M.M., Musio A. (2020). Cornelia de Lange syndrome: from molecular diagnosis to therapeutic approach. J. Med. Genet..

[307] Vega H., Waisfisz Q., Gordillo M., Sakai N., Yanagihara I., Yamada M., van Gosliga D., Kayserili H., Xu C., Ozono K., Wang Jabs E., Inui K., Joenje H. (2005). Roberts syndrome is caused by mutations in ESCO2, a human homolog of yeast ECO1 that is essential for the establishment of sister chromatid cohesion. Nat. Genet..

[308] Ivanov D., Schleiffer A., Eisenhaber F., Mechtler K., Haering C.H., Nasmyth K. (2002). Eco1 is a novel acetyltransferase that can acetylate proteins involved in cohesion. Curr. Biol..

[309] Nora E.P., Lajoie B.R., Schulz E.G., Giorgetti L., Okamoto I., Servant N., Piolot T., van Berkum N.L., Meisig J., Sedat J., Gribnau J., Barillot E., Blüthgen N., Dekker J., Heard E. (2012). Spatial partitioning of the regulatory landscape of the X-inactivation centre. Nature.

[310] Dekker J., Marti-Renom M.A., Mirny L.A. (2013). Exploring the three-dimensional organization of genomes: interpreting chromatin interaction data. Nat. Rev. Genet..

[311] Yu M., Ren B. (2017). The three-dimensional organization of mammalian genomes. Annu. Rev. Cell Dev. Biol..

[312] Eagen K.P., Hartl T.A., Kornberg R.D. (2015). Stable chromosome condensation revealed by chromosome conformation capture. Cell.

[313] Painter T.S. (1933). A new method for the study of chromosome rearrangements and plotting of chromosome maps. Science.

[314] Merkenschlager M., Nora E.P. (2016). CTCF and cohesin in genome folding and transcriptional gene regulation. Annu. Rev. Genom. Hum. Genet..

[315] Serrano D., Cordero G., Kawamura R., Sverzhinsky A., Sarker M., Roy S., Malo C., Pascal J.M., Marko J.F., D’Amours D. (2020). The Smc5/6 core complex is a structure-specific DNA binding and compacting machine. Mol. Cell.

[316] Gutierrez-Escribano P., Hormeño S., Madariaga-Marcos J., Solé-Soler R., O’Reilly F.J., Morris K., Aicart-Ramos C., Aramayo R., Montoya A., Kramer H., Rappsilber J., Torres-Rosell J., Moreno-Herrero F., Aragon L. (2020). Purified Smc5/6 complex exhibits DNA substrate recognition and compaction. Mol. Cell.

[317] Venegas A.B., Natsume T., Kanemaki M., Hickson I.D. (2020). Inducible degradation of the human SMC5/6 complex reveals an essential role only during interphase. Cell Rep..

[318] Lee Y.M., Lee S., Lee E., Shin H., Hahn H., Choi W., Kim W. (2001). Human kinesin superfamily member 4 is dominantly localized in the nuclear matrix and is associated with chromosomes during mitosis. Biochem. J..

[319] Mazumdar M., Misteli T. (2005). Chromokinesins: multitalented players in mitosis. Trends Cell Biol..

[320] Poonperm R., Takata H., Uchiyama S., Fukui K. (2017). Interdependency and phosphorylation of KIF4 and condensin I are essential for organization of chromosome scaffold. PLoS One.

[321] Poser E., Caous R., Gruneberg U., Barr F.A. (2019). Aurora A promotes chromosome congression by activating the condensin-dependent pool of KIF4A. J. Cell Biol..

[322] Takahashi M., Tanaka K., Wakai T., Hirota T. (2016). Phosphoproteomic analysis of human mitotic chromosomes identified a chromokinesin KIF4A. Biomed. Res..

[323] Sekine Y., Okada Y., Noda Y., Kondo S., Aizawa H., Takemura R., Hirokawa N. (1994). A novel microtubule-based motor protein (KIF4) for organelle transports, whose expression is regulated developmentally. J. Cell Biol..

[324] Bringmann H. (2004). A kinesin-like motor inhibits microtubule dynamic instability. Science.

[325] Kurasawa Y., Earnshaw W.C., Mochizuki Y., Dohmae N., Todokoro K. (2004). Essential roles of KIF4 and its binding partner PRC1 in organized central spindle midzone formation. EMBO J..

[326] Hu C.K., Coughlin M., Field C.M., Mitchison T.J. (2011). KIF4 regulates midzone length during cytokinesis. Curr. Biol..

[327] Hannabuss J., Lera-Ramirez M., Cade N.I., Fourniol F.J., Nédélec F., Surrey T. (2019). Self-organization of minimal anaphase spindle midzone bundles. Curr. Biol..

[328] Wu G., Zhou L., Khidr L., Guo X.E., Kim W., Lee Y.M., Krasieva T., Chen P.L. (2008). A novel role of the chromokinesin Kif4A in DNA damage response. Cell Cycle.

[329] Wan Q., Shen Y., Zhao H., Wang B., Zhao L., Zhang Y., Bu X., Wan M., Shen C. (2019). Impaired DNA double-strand breaks repair by kinesin family member 4A inhibition renders human H1299 non-small-cell lung cancer cells sensitive to cisplatin. J. Cell Physiol..

[330] Song X., Zhang T., Wang X., Liao X., Han C., Yang C., Su K., Cao W., Gong Y., Chen Z., Han Q., Li J. (2018). Distinct diagnostic and prognostic values of kinesin family member genes expression in patients with breast cancer. Med. Sci. Monit..

[331] Trevino V. (2019). Integrative genomic analysis identifies associations of molecular alterations to APOBEC and BRCA1/2 mutational signatures in breast cancer. Mol. Genet. Genom. Med..

[332] Chen T., Yang S., Xu J., Lu W., Xie X. (2020). Transcriptome sequencing profiles of cervical cancer tissues and SiHa cells. Funct. Integr. Genom..

[333] Sheng L., Hao S.L., Yang W.X., Sun Y. (2018). The multiple functions of kinesin-4 family motor protein KIF4 and its clinical potential. Gene.

[334] Mazumdar M., Sundareshan S., Misteli T. (2004). Human chromokinesin KIF4A functions in chromosome condensation and segregation. J. Cell Biol..

[335] Geiman T.M. (2004). Isolation and characterization of a novel DNA methyltransferase complex linking DNMT3B with components of the mitotic chromosome condensation machinery. Nucleic Acids Res..

[336] Mazumdar M., Sung M.H., Misteli T. (2011). Chromatin maintenance by a molecular motor protein. Nucleus.

[337] Dong Z., Zhu C., Zhan Q., Jiang W. (2018). Cdk phosphorylation licenses Kif4A chromosome localization required for early mitotic progression. J. Mol. Cell Biol..

[338] Kakui Y., Uhlmann F. (2017). Building chromosomes without bricks. Science.

[339] Scovassl A.I., Mariani C., Negroni M., Negri C., Bertazzoni U. (1993). ADP-ribosylation of nonhistone proteins in HeLa cells: modification of DNA topoisomerase II. Exp. Cell Res..

[340] Darby M.K., Schmitt B., Jongstra-Bilen J., Vosberg H.P. (1985). Inhibition of calf thymus type II DNA topoisomerase by poly(ADP-ribosylation). EMBO J..

[341] Heck M.M.S., Hittelman W.N., Earnshaw W.C. (1989). In vivo phosphorylation of the 170-kDa form of eukaryotic DNA topoisomerase II. J. Biol. Chem..

[342] Saijo M., Ui M., Enomoto T. (1992). Growth state and cell cycle dependent phosphorylation of DNA topoisomerase IIin Swiss 3T3 cells. Biochemistry.

[343] Taagepera S., Rao P.N., Drake F.H., Gorbsky G.J. (1993). DNA topoisomerase IIa is the major chromosome protein recognized by the mitotic phosphoprotein antibody MPM-2. Proc. Nat. Acad. Sci..

[344] Wells N.J., Hickson I.D. (1995). Human topoisomerase II alpha is phosphorylated in a cell-cycle phase-dependent manner by a proline-directed kinase. Eur. J. Biochem..

[345] Daum J.R., Gorbsky G.J. (1998). Casein kinase II catalyzes a mitotic phosphorylation on threonine 1342 of human DNA topoisomerase IIalpha, which is recognized by the 3F3/2 phosphoepitope antibody. J. Biol. Chem..

[346] Ishida R., Iwai M., Marsh K.L., Austin C.A., Yano T., Shibata M., Nozaki N., Hara A. (1996). Threonine 1342 in human topoisomerase IIalpha is phosphorylated throughout the cell cycle. J. Biol. Chem..

[347] Escargueil A.E., Plisov S.Y., Filhol O., Cochet C., Larsen A.K. (2000). Mitotic phosphorylation of DNA topoisomerase II{alpha} by protein kinase CK2 creates the MPM-2 phosphoepitope on Ser-1469. J. Biol. Chem..

[348] Ackerman P., Glover C.V.C., Osheroff N. (1985). Phosphorylation of DNA topoisomerase II by casein kinase II: modulation of eukaryotic topoisomerase II activity in vitro. Proc. Natl. Acad. Sci..

[349] Sahyoun N., Wolf M., Besterman J., Hsieh T., Sander M., LeVine H., Chang K.J., Cuatrecasas P. (1986). Protein kinase C phosphorylates topoisomerase II: topoisomerase activation and its possible role in phorbol ester-induced differentiation of HL-60 cells. Proc. Natl. Acad. Sci..

[350] Ackerman P., Glover C.V.C., Osheroff N. (1988). Phosphorylation of DNA topoisomerase II in vivo and in total homogenates of Drosophila Kc cells. The role of casein kinase II. J. Biol. Chem..

[351] Cardenas M.E., Dang Q., Glover C.V., Gasser S.M. (1992). Casein kinase II phosphorylates the eukaryote-specific C-terminal domain oftopoisomerase II in vivo. EMBO J..

[352] Cardenas M.E., Gasser S.M. (1993). Casein kinase II copurifies with yeast topoisomerase II and reactivates the dephosphorylated enzyme. J. Cell Sci..

[353] Shiozaki K., Yanagida M. (1992). Functional dissection of the phosphorylated termini of fission yeast DNA topoisomerase II. J. Cell Biol..

[354] Cardenas M.E., Gasser S.M. (1993). Regulation of topoisomerase II by phosphorylation: a role for casein kinase II. J. Cell Sci..

[355] Dang Q., Alghisi G.-C., Gasser S.M. (1994). Phosphorylation of the C-terminal domain of yeast topoisomerase II by casein kinase II affects DNA-protein interaction. J. Mol. Biol..

[356] Dickey J.S., Osheroff N. (2005). Impact of the C-terminal domain of topoisomerase IIalpha on the DNA cleavage activity of the human enzyme. Biochemistry.

[357] Yoshida M.M., Ting L., Gygi S.P., Azuma Y. (2016). SUMOylation of DNA topoisomerase IIalpha regulates histone H3 kinase Haspin and H3 phosphorylation in mitosis. J. Cell Biol..

[358] Pandey N., Keifenheim D., Yoshida M.M., Hassebroek V.A., Soroka C., Azuma Y., Clarke D.J. (2020). Topoisomerase II SUMOylation activates a metaphase checkpoint via Haspin and Aurora B kinases. J. Cell Biol..

[359] Takemoto A., Kimura K., Yokoyama S., Hanaoka F. (2004). Cell cycle-dependent phosphorylation, nuclear localization, and activation of human condensin. J. Biol. Chem..

[360] Abe S., Nagasaka K., Hirayama Y., Kozuka-Hata H., Oyama M., Aoyagi Y., Obuse C., Hirota T. (2011). The initial phase of chromosome condensation requires Cdk1-mediated phosphorylation of the CAP-D3 subunit of condensin II. Genes Dev..

[361] Giet R., Glover D.M. (2001). Drosophila aurora B kinase is required for histone H3 phosphorylation and condensin recruitment during chromosome condensation and to organize the central spindle during cytokinesis. J. Cell Biol..

[362] Takemoto A., Murayama A., Katano M., Urano T., Furukawa K., Yokoyama S., Yanagisawa J., Hanaoka F., Kimura K. (2007). Analysis of the role of Aurora B on the chromosomal targeting of condensin I. Nucleic Acids Res..

[363] Lipp J.J., Hirota T., Poser I., Peters J.M. (2007). Aurora B controls the association of condensin I but not condensin II with mitotic chromosomes. J. Cell Sci..

[364] Murphy L.A., Sarge K.D. (2008). Phosphorylation of CAP-G is required for its chromosomal DNA localization during mitosis. Biochem. Biophys. Res. Commun..

[365] Nakazawa N., Mehrotra R., Ebe M., Yanagida M. (2011). Condensin phosphorylated by the Aurora-B-like kinase Ark1 is continuously required until telophase in a mode distinct from Top2. J. Cell Sci..

[366] Tada K., Susumu H., Sakuno T., Watanabe Y. (2011). Condensin association with histone H2A shapes mitotic chromosomes. Nature.

[367] Kagami Y., Nihira K., Wada S., Ono M., Honda M., Yoshida K. (2014). Mps1 phosphorylation of condensin II controls chromosome condensation at the onset of mitosis. J. Cell Biol..

[368] Kagami Y., Ono M., Yoshida K. (2017). Plk1 phosphorylation of CAP-H2 triggers chromosome condensation by condensin II at the early phase of mitosis. Sci. Rep..

[369] Zhang T., Si-Hoe S.L., Hudson D.F., Surana U. (2016). Condensin recruitment to chromatin is inhibited by Chk2 kinase in response to DNA damage. Cell Cycle.

[370] Takemoto A., Kimura K., Yanagisawa J., Yokoyama S., Hanaoka F. (2006). Negative regulation of condensin I by CK2-mediated phosphorylation. EMBO J..

[371] Akai Y., Kanai R., Nakazawa N., Ebe M., Toyoshima C., Yanagida M. (2014). ATPase-dependent auto-phosphorylation of the open condensin hinge diminishes DNA binding. Open Biol..

[372] Kimura K. (1998). Phosphorylation and activation of 13S condensin by Cdc2 in vitro. Science.

[373] Sutani T., Yuasa T., Tomonaga T., Dohmae N., Takio K., Yanagida M. (1999). Fission yeast condensin complex: essential roles of non-SMC subunits for condensation and Cdc2 phosphorylation of Cut3/SMC4. Genes Dev..

[374] St-Pierre J., Douziech M., Bazile F., Pascariu M., Bonneil É., Sauvé V., Ratsima H., D’Amours D. (2009). Polo kinase regulates mitotic chromosome condensation by hyperactivation of condensin DNA supercoiling activity. Mol. Cell.

[375] Takata H., Madung M., Katoh K., Fukui K. (2018). Cdk1-dependent phosphorylation of KIF4A at S1186 triggers lateral chromosome compaction during early mitosis. PLoS One.

[376] Nunes Bastos, R., et al (2013). Aurora B suppresses microtubule dynamics and limits central spindle size by locally activating KIF4A. J. Cell Biol..

[377] Bastos R.N., Cundell M.J., Barr F.A. (2014). KIF4A and PP2A-B56 form a spatially restricted feedback loop opposing Aurora B at the anaphase central spindle. J. Cell Biol..

[378] Li Q.R., Yan X.M., Guo L., Li J., Zang Y. (2018). AMPK regulates anaphase central spindle length by phosphorylation of KIF4A. J. Mol. Cell Biol..

[379] Riggs A.D. (1990). DNA methylation and late replication probably aid cell memory, and type I DNA reeling could aid chromosome folding and enhancer function. Philos. Trans. R. Soc. Lond. B Biol. Sci..

[380] Nasmyth K. (2001). Disseminating the genome: joining, resolving, and separating sister chromatids during mitosis and meiosis. Annu. Rev. Genet..

[381] Goloborodko A., Imakaev M.V., Marko J.F., Mirny L. (2016). Compaction and segregation of sister chromatids via active loop extrusion. Elife.

[382] Fudenberg G., Imakaev M., Lu C., Goloborodko A., Abdennur N., Mirny L.A. (2016). Formation of chromosomal domains by loop extrusion. Cell Rep..

[383] Goloborodko A., Marko J.F., Mirny L.A. (2016). Chromosome compaction by active loop extrusion. Biophys. J..

[384] Fudenberg G., Abdennur N., Imakaev M., Goloborodko A., Mirny L.A. (2017). Emerging evidence of chromosome folding by loop extrusion. Cold Spring Harb. Symp. Quant. Biol..

[385] Ganji M., Shaltiel I.A., Bisht S., Kim E., Kalichava A., Haering C.H., Dekker C. (2018). Real-time imaging of DNA loop extrusion by condensin. Science.

[386] Davidson I.F., Bauer B., Goetz D., Tang W., Wutz G., Peters J.M. (2019). DNA loop extrusion by human cohesin. Science.

[387] Kim Y., Shi Z., Zhang H., Finkelstein I.J., Yu H. (2019). Human cohesin compacts DNA by loop extrusion. Science.

[388] Barrington C., Finn R., Hadjur S. (2017). Cohesin biology meets the loop extrusion model. Chromosome Res..

[389] Banigan E.J., Mirny L.A. (2020). Loop extrusion: theory meets single-molecule experiments. Curr. Opin. Cell Biol..

[390] Terakawa T., Bisht S., Eeftens J.M., Dekker C., Haering C.H., Greene E.C. (2017). The condensin complex is a mechanochemical motor that translocates along DNA. Science.

[391] Kim E., Kerssemakers J., Shaltiel I.A., Haering C.H., Dekker C. (2020). DNA-loop extruding condensin complexes can traverse one another. Nature.

[392] Kong M., Cutts E.E., Pan D., Beuron F., Kaliyappan T., Xue C., Morris E.P., Musacchio A., Vannini A., Greene E.C. (2020). Human condensin I and II drive extensive ATP-dependent compaction of nucleosome-bound DNA. Mol. Cell.

[393] Golfier S., Quail T., Kimura H., Brugués J. (2020). Cohesin and condensin extrude DNA loops in a cell cycle-dependent manner. Elife.

[394] Hakes D.J., Berezney R. (1991). DNA binding properties of the nuclear matrix and individual nuclear matrix proteins. Evidence for salt-resistant DNA binding sites. J. Biol. Chem..

[395] Jackson D., Dolle A., Robertson G., Cook P. (1992). The attachments of chromatin loops to the nucleoskeleton. Cell Biol. Int. Rep..

[396] Dickinson L., Joh T., Kohwi Y., Kohwi-Shigematsu T. (1992). A tissue-specific MAR/SAR DNA-binding protein with unusual binding site recognition. Cell.

[397] Bode J., Schlake T., Rios-Ramirez M., Mielke C., Stengert M., Kay V., Klehr-Wirth D. (1995). Scaffold/matrix-attached regions: structural properties creating transcriptionally active loci. Int. Rev. Cytol..

[398] Tsutsui K., Tsutsui K., Muller M.T. (1988). The nuclear scaffold exhibits DNA-binding sites selective for supercoiled DNA. J. Biol. Chem..

[399] Homberger H.P. (1989). Bent DNA is a structural feature of scaffold-attached regions in Drosophila melanogaster interphase nuclei. Chromosoma.

[400] Ludérus M.E., den Blaauwen J.L., de Smit O.J., Compton D.A., van Driel R. (1994). Binding of matrix attachment regions to lamin polymers involves single-stranded regions and the minor groove. Mol. Cell Biol..

[401] Rubio E.D., Reiss D.J., Welcsh P.L., Disteche C.M., Filippova G.N., Baliga N.S., Aebersold R., Ranish J.A., Krumm A. (2008). CTCF physically links cohesin to chromatin. Proc. Natl. Acad. Sci. USA.

[402] Parelho V., Hadjur S., Spivakov M., Leleu M., Sauer S., Gregson H.C., Jarmuz A., Canzonetta C., Webster Z., Nesterova T., Cobb B.S., Yokomori K., Dillon N., Aragon L., Fisher A.G., Merkenschlager M. (2008). Cohesins functionally associate with CTCF on mammalian chromosome arms. Cell.

[403] Stedman W., Kang H., Lin S., Kissil J.L., Bartolomei M.S., Lieberman P.M. (2008). Cohesins localize with CTCF at the KSHV latency control region and at cellular c-myc and H19/Igf2 insulators. EMBO J..

[404] Wendt K.S., Yoshida K., Itoh T., Bando M., Koch B., Schirghuber E., Tsutsumi S., Nagae G., Ishihara K., Mishiro T., Yahata K., Imamoto F., Aburatani H., Nakao M., Imamoto N., Maeshima K., Shirahige K., Peters J.M. (2008). Cohesin mediates transcriptional insulation by CCCTC-binding factor. Nature.

[405] Schmidt C.K., Brookes N., Uhlmann F. (2009). Conserved features of cohesin binding along fission yeast chromosomes. Genome Biol..

[406] Ocampo-Hafalla M., Muñoz S., Samora C.P., Uhlmann F. (2016). Evidence for cohesin sliding along budding yeast chromosomes. Open Biol..

[407] Davidson I.F., Goetz D., Zaczek M.P., Molodtsov M.I., Huis in ’t Veld P.J., Weissmann F., Litos G., Cisneros D.A., Ocampo‐Hafalla M., Ladurner R., Uhlmann F., Vaziri A., Peters J.M. (2016). Rapid movement and transcriptional re-localization of human cohesin on DNA. EMBO J..

[408] Dixon J.R., Selvaraj S., Yue F., Kim A., Li Y., Shen Y., Hu M., Liu J.S., Ren B. (2012). Topological domains in mammalian genomes identified by analysis of chromatin interactions. Nature.

[409] Oomen M.E., Hedger A.K., Watts J.K., Dekker J. (2020). Detecting chromatin interactions between and along sister chromatids with SisterC. Nat. Methods.

[410] Nora E.P., Goloborodko A., Valton A.L., Gibcus J.H., Uebersohn A., Abdennur N., Dekker J., Mirny L.A., Bruneau B.G. (2017). Targeted degradation of CTCF decouples local insulation of chromosome domains from genomic compartmentalization. Cell.

[411] Mirkovitch J., Mirault M., Laemmli U.K. (1984). Organization of the higher-order chromatin loop: specific DNA attachment sites on nuclear scaffold. Cell.

[412] Mirkovitch J., Gasser S.M., Laemmli U.K. (1988). Scaffold attachment of DNA loops in metaphase chromosomes. J. Mol. Biol..

[413] Gasser S.M., Laemmli U.K. (1986). The organisation of chromatin loops: characterization of a scaffold attachment site. EMBO J..

[414] Adachi Y., Kas E., Laemmli U.K. (1989). Preferential, cooperative binding of DNA topoisomerase II to scaffold-associated regions. EMBO J..

[415] Laemmli U.K., Käs E., Poljak L., Adachi Y. (1992). Scaffold-associated regions: cis-acting determinants of chromatin structural loops and functional domains. Curr. Opin. Genet. Dev..

[416] Kim J.H., Zhang T., Wong N.C., Davidson N., Maksimovic J., Oshlack A., Earnshaw W.C., Kalitsis P., Hudson D.F. (2013). Condensin I associates with structural and gene regulatory regions in vertebrate chromosomes. Nat. Commun..

[417] Dowen J.M., Bilodeau S., Orlando D.A., Hübner M.R., Abraham B.J., Spector D.L., Young R.A. (2013). Multiple structural maintenance of chromosome complexes at transcriptional regulatory elements. Stem Cell Rep..

[418] Alipour E., Marko J.F. (2012). Self-organization of domain structures by DNA-loop-extruding enzymes. Nucleic Acids Res..

[419] Heermann D.W. (2011). Physical nuclear organization: loops and entropy. Curr. Opin. Cell Biol..

[420] Cheng T.M., Heeger S., Chaleil R.A., Matthews N., Stewart A., Wright J., Lim C., Bates P.A., Uhlmann F. (2015). A simple biophysical model emulates budding yeast chromosome condensation. Elife.

[421] Gerguri T., Fu X., Kakui Y., Khatri B.S., Barrington C., Bates P.A., Uhlmann F. (2021). Comparison of loop extrusion and diffusion capture as mitotic chromosome formation pathways in fission yeast. Nucleic Acids Res..

[422] Dekker J. (2002). Capturing chromosome conformation. Science.

[423] Splinter E., de Wit E., Nora E.P., Klous P., van de Werken H.J.G., Zhu Y., Kaaij L.J.T., van IJcken W., Gribnau J., Heard E., de Laat W. (2011). The inactive X chromosome adopts a unique three-dimensional conformation that is dependent on Xist RNA. Genes Dev..

[424] Lieberman-Aiden E., van Berkum N.L., Williams L., Imakaev M., Ragoczy T., Telling A., Amit I., Lajoie B.R., Sabo P.J., Dorschner M.O., Sandstrom R., Bernstein B., Bender M.A., Groudine M., Gnirke A., Stamatoyannopoulos J., Mirny L.A., Lander E.S., Dekker J. (2009). Comprehensive mapping of long-range interactions reveals folding principles of the human genome. Science.

[425] de Wit E., de Laat W. (2012). A decade of 3C technologies: insights into nuclear organization. Genes Dev..

[426] Sati S., Cavalli G. (2017). Chromosome conformation capture technologies and their impact in understanding genome function. Chromosoma.

[427] Holwerda S., de Laat W. (2012). Chromatin loops, gene positioning, and gene expression. Front. Genet..

[428] Beagrie R.A., Pombo A. (2017). Cell cycle: continuous chromatin changes. Nature.

[429] Wang C.Y., Jégu T., Chu H.P., Oh H.J., Lee J.T. (2018). SMCHD1 merges chromosome compartments and assists formation of super-structures on the inactive X. Cell.

[430] Oomen M.E., Hansen A.S., Liu Y., Darzacq X., Dekker J. (2019). CTCF sites display cell cycle-dependent dynamics in factor binding and nucleosome positioning. Genome Res..

[431] Bishop A.C., Shah K., Liu Y., Witucki L., Kung C., Shokat K.M. (1998). Design of allele-specific inhibitors to probe protein kinase signaling. Curr. Biol..

[432] Bishop A.C., Ubersax J.A., Petsch D.T., Matheos D.P., Gray N.S., Blethrow J., Shimizu E., Tsien J.Z., Schultz P.G., Rose M.D., Wood J.L., Morgan D.O., Shokat K.M. (2000). A chemical switch for inhibitor-sensitive alleles of any protein kinase. Nature.

[433] Papa F.R. (2003). Bypassing a kinase activity with an ATP-competitive drug. Science.

[434] Hochegger H., Dejsuphong D., Sonoda E., Saberi A., Rajendra E., Kirk J., Hunt T., Takeda S. (2007). An essential role for Cdk1 in S phase control is revealed via chemical genetics in vertebrate cells. J. Cell Biol..

[435] Nishimura K., Fukagawa T., Takisawa H., Kakimoto T., Kanemaki M. (2009). An auxin-based degron system for the rapid depletion of proteins in nonplant cells. Nat. Methods.

[436] Lenart P., Ellenberg J. (2006). Monitoring the permeability of the nuclear envelope during the cell cycle. Methods.

[437] Lénárt P., Rabut G., Daigle N., Hand A.R., Terasaki M., Ellenberg J. (2003). Nuclear envelope breakdown in starfish oocytes proceeds by partial NPC disassembly followed by a rapidly spreading fenestration of nuclear membranes. J. Cell Biol..

[438] Laurell E., Beck K., Krupina K., Theerthagiri G., Bodenmiller B., Horvath P., Aebersold R., Antonin W., Kutay U. (2011). Phosphorylation of Nup98 by multiple kinases is crucial for NPC disassembly during mitotic entry. Cell.

[439] Walther N., Hossain M.J., Politi A.Z., Koch B., Kueblbeck M., Ødegård-Fougner Ø., Lampe M., Ellenberg J. (2018). A quantitative map of human Condensins provides new insights into mitotic chromosome architecture. J. Cell Biol..

[440] Roberge M., Th’ng J., Hamaguchi J., Bradbury E.M. (1990). The topoisomerase II inhibitor VM-26 induces marked changes in histone H1 kinase activity, histones H1 and H3 phosphorylation, and chromosome condensation in G2 phase and mitotic BHK cells. J. Cell Biol..

[441] Vagnarelli P., Hudson D.F., Ribeiro S.A., Trinkle-Mulcahy L., Spence J.M., Lai F., Farr C.J., Lamond A.I., Earnshaw W.C. (2006). Condensin and Repo-Man-PP1 co-operate in the regulation of chromosome architecture during mitosis. Nat. Cell Biol..

[442] Samoshkin A., Arnaoutov A., Jansen L.E.T., Ouspenski I., Dye L., Karpova T., McNally J., Dasso M., Cleveland D.W., Strunnikov A. (2009). Human condensin function is essential for centromeric chromatin assembly and proper sister kinetochore orientation. PLoS One.

[443] Giménez-Abián J.F., Clarke D.J., Devlin J., Giménez-Abián M.I., De la Torre C., Johnson R.T., Mullinger A.M., Downes C.S. (2000). Premitotic chromosome individualization in mammalian cells depends on topoisomerase II activity. Chromosoma.

[444] Stanyte R., Nuebler J., Blaukopf C., Hoefler R., Stocsits R., Peters J.M., Gerlich D.W. (2018). Dynamics of sister chromatid resolution during cell cycle progression. J. Cell Biol..

[445] Mitter M., Gasser C., Takacs Z., Langer C.C.H., Tang W., Jessberger G., Beales C.T., Neuner E., Ameres S.L., Peters J.M., Goloborodko A., Micura R., Gerlich D.W. (2020). Conformation of sister chromatids in the replicated human genome. Nature.

[446] Mitter M., Gerlich D.W. (2021). Mapping sister chromatid conformation in replicated chromosomes. Trends Biochem. Sci..

[447] St-Pierre J., Douziech M., Bazile F., Pascariu M., Bonneil É., Sauvé V., Ratsima H., D’Amours D. (2009). Polo kinase regulates mitotic chromosome condensation by hyperactivation of condensin DNA supercoiling activity. Mol. Cell.

[448] Strick R., Strissel P.L., Gavrilov K., Levi-Setti R. (2001). Cation-chromatin binding as shown by ion microscopy is essential for the structural integrity of chromosomes. J. Cell Biol..

[449] Phengchat R., Takata H., Morii K., Inada N., Murakoshi H., Uchiyama S., Fukui K. (2016). Calcium ions function as a booster of chromosome condensation. Sci. Rep..

[450] Maeshima K., Matsuda T., Shindo Y., Imamura H., Tamura S., Imai R., Kawakami S., Nagashima R., Soga T., Noji H., Oka K., Nagai T. (2018). A transient rise in free Mg(2+) ions released from ATP-Mg hydrolysis contributes to mitotic chromosome condensation. Curr. Biol..

[451] Patel A., Malinovska L., Saha S., Wang J., Alberti S., Krishnan Y., Hyman A.A. (2017). ATP as a biological hydrotrope. Science.

[452] Bradbury E.M., Inglis R.J., Matthews H.R., Sarner N. (1973). Phosphorylation of very-lysine-rich histone in Physarum polycephalum. Correlation with chromosome condensation. Eur. J. Biochem..

[453] Th’ng J.P., Guo X.W., Swank R.A., Crissman H.A., Bradbury E.M. (1994). Inhibition of histone phosphorylation by staurosporine leads to chromosome decondensation. J. Biol. Chem..

[454] Paulson J.R., Vander Mause E.R. (2013). Calyculin A induces prematurely condensed chromosomes without histone H1 phosphorylation in mammalian G1-phase cells. Adv. Biol. Chem..

[455] Lowary P.T., Widom J. (1998). New DNA sequence rules for high affinity binding to histone octamer and sequence-directed nucleosome positioning. J. Mol. Biol..

[456] Thåström A., Lowary P.T., Widlund H.R., Cao H., Kubista M., Widom J. (1999). Sequence motifs and free energies of selected natural and non-natural nucleosome positioning DNA sequences. J. Mol. Biol..

[457] Rodriguez-Collazo P., Leuba S.H., Zlatanova J. (2009). Robust methods for purification of histones from cultured mammalian cells with the preservation of their native modifications. Nucleic Acids Res..

[458] Leidecker O., Bonfiglio J.J., Colby T., Zhang Q., Atanassov I., Zaja R., Palazzo L., Stockum A., Ahel I., Matic I. (2016). Serine is a new target residue for endogenous ADP-ribosylation on histones. Nat. Chem. Biol..

[459] Zhiteneva A., Bonfiglio J.J., Makarov A., Colby T., Vagnarelli P., Schirmer E.C., Matic I., Earnshaw W.C. (2017). Mitotic post-translational modifications of histones promote chromatin compaction in vitro. Open Biol..

[460] Kleinschmidt A.K., Lang D., Zahn R.K. (1960). Darstellung molekularer Fäden von Desoxyribonucleinsäuren. Naturwissenschaften.

[461] Anderson D.E., Losada A., Erickson H.P., Hirano T. (2002). Condensin and cohesin display different arm conformations with characteristic hinge angles. J. Cell Biol..

[462] Yoshimura S.H., Hizume K., Murakami A., Sutani T., Takeyasu K., Yanagida M. (2002). Condensin architecture and interaction with DNA: regulatory non-SMC subunits bind to the head of SMC heterodimer. Curr. Biol..

[463] Ryu J.K., Katan A.J., van der Sluis E.O., Wisse T., de Groot R., Haering C.H., Dekker C. (2020). The condensin holocomplex cycles dynamically between open and collapsed states. Nat. Struct. Mol. Biol..

